# Serum and Liver Lipidome Following Empagliflozin Administration for Six Months in a Fast Food Diet Mouse Model

**DOI:** 10.3390/ijms26199273

**Published:** 2025-09-23

**Authors:** Evangelia S. Makri, Thomai Mouskeftara, Helen Gika, Konstantinos Xanthopoulos, Eleftheria Makri, Panagiotis Mavrommatis-Parasidis, Anastasia Tsingotjidou, Angeliki Cheva, Antonis Goulas, Stergios A. Polyzos

**Affiliations:** 1 First Laboratory of Pharmacology, School of Medicine, Aristotle University of Thessaloniki, 54124 Thessaloniki, Greeceagoulas@auth.gr (A.G.); 2 Laboratory of Forensic Medicine & Toxicology, Department of Medicine, Aristotle University of Thessaloniki, 54124 Thessaloniki, Greecegkikae@auth.gr (H.G.); 3 Biomic AUTh, Center for Interdisciplinary Research and Innovation (CIRI-AUTH), Balkan Center B1.4, 57001 Thessaloniki, Greece; 4 Laboratory of Pharmacology, School of Pharmacy, Aristotle University of Thessaloniki, 54124 Thessaloniki, Greece; xantho@pharm.auth.gr; 5 Institute of Applied Biosciences, Centre for Research and Technology, 57001 Thessaloniki, Greece; 6 Laboratory of Anatomy, Histology & Embryology, School of Veterinary Medicine, Aristotle University of Thessaloniki, 54124 Thessaloniki, Greeceastsing@vet.auth.gr (A.T.); 7 Department of Pathology, School of Medicine, Aristotle University of Thessaloniki, 54124 Thessaloniki, Greece; antacheva@auth.gr

**Keywords:** empagliflozin, hepatic steatosis, fast food diet, untargeted lipidomics, metabolic dysfunction-associated steatotic liver disease, mouse model, nonalcoholic fatty liver disease

## Abstract

Empagliflozin is a sodium–glucose co-transporter inhibitor approved for the treatment of type 2 diabetes mellitus. The aim of this study was the 6-month effect of empagliflozin on serum and liver lipidome in C57BL/6J mice fed on a fast food diet (FFD). Three groups were studied; two of them fed on FFD, one with empagliflozin (EMPA group), and another without empagliflozin (FFD group); the third group fed on a chow diet and served as the control group (CD group). Following untargeted lipidomic analysis, the FFD and EMPA groups displayed largely similar serum lipid profiles, characterized by elevated levels in the majority of identified lipids, compared with the CD group, particularly glycerophospholipids. For instance, phosphatidylcholine (PC) 34:1 and phosphatidylinositol (PI) 38:3 increased in the FFD compared with the CD group (both *p* < 0.001, fold change 2.4 and 17.6, respectively) with comparable increases observed in the EMPA group. Hepatic lipid profiles varied more significantly between groups. For example, PC 34:1 was increased in the FFD and in the EMPA compared with the CD group (both *p* < 0.001, fold change 1.7 and 1.6, respectively), whereas PC 32:0 was decreased in the FFD group and in the EMPA group compared with the CD group (both *p* < 0.001, fold change 0.6 and 0.5, respectively). FFD appears to have a more substantial impact on lipidomic profiles compared with the preventive empagliflozin effect. Notably, the concentration of lysophosphatidylcholine (LPC) 22:6 was significantly reduced in the EMPA compared with the FFD group (*p* < 0.001, fold change 1.4). Interestingly, several glycerophospholipids, including PC 34:1, PC 35:1, PC 36:3, PC 38:4, PI 34:2 and PI 38:3, increased in both serum and hepatic tissues of the FFD and EMPA groups compared with the CD group. In conclusion, limited differences in the lipidomic profile were observed in the EMPA compared with the FFD group (e.g., LPC 22:6). However, both the EMPA and FFD groups showed distinct lipidomic profiles compared with the CD group.

## 1. Introduction

Metabolic dysfunction-associated steatotic liver disease (MASLD) [1], previously known as nonalcoholic fatty liver disease (NAFLD), is a highly prevalent disease, affecting approximately 30% of the global adult population [2], with very limited pharmacological options [3]. The increasing prevalence of MASLD worldwide correlates with the growing rates of obesity and type 2 diabetes mellitus (T2DM) [4], thus MASLD prevalence can rise up to 65% in individuals with T2DM [2]. The pathogenesis of MASLD is multifactorial and not fully elucidated. According to the “multiple-hit” hypothesis, numerous pathogenic factors may act concurrently and continuously or intermittently during the pathogenesis of MASLD in each affected individual [5]. Adopting this pathogenic “multiple-hit” model could have crucial therapeutic implications, as different pathogenic factors need to be managed in each patient with MASLD in a personalized approach [4,6].

Beyond obesity and T2DM, MASLD is also closely associated with other components of the metabolic syndrome (MetS), such as dyslipidemia and arterial hypertension [7]. Insulin resistance is a crucial mechanistic link between MASLD and MetS components, especially glucose dysmetabolism [7]. Glucose dysmetabolism in the setting of MetS is characterized by impaired insulin secretion, and inappropriate insulin response, leading to insulin resistance [8]. Notably, some authors have supported that dyslipidemia, which commonly coexists with T2DM, may precede hyperglycemia [8]. In this regard, specific lipid metabolites, such as certain cholesterol esters, were shown to positively correlate with clinical parameters related to glucose and lipid metabolism in patients with T2DM [8]. It was also supported that lipid dysmetabolism, including alterations in triacylglycerols, phosphatidylcholines, and sphingomyelins, seem to contribute to the progression of MASLD [9]. Chronic inflammation, which interacts with insulin resistance and dyslipidemia, also contributes to the progression of MASLD [10]. Noteworthy, the spleen has emerged as a critical modulator of this chronic, low-grade inflammatory state, mostly through the liver–spleen axis. As an immune reservoir and an amplifier of inflammation, the spleen releases pro-inflammatory cytokines like tumor necrosis factor-α, interleukin (IL)-6, and IL-1β, which worsen insulin resistance and hepatic lipid accumulation [11]. Other authors supported that splenic monocytes migrate from the spleen into the liver, in which they differentiate into macrophages; subsequently, these macrophages activate hepatic stellate cells, thus promoting hepatic fibrogenesis [12]. In clinical terms, spleen volume was associated with MASLD and related fibrosis [13]. More specifically, spleen enlargement was independently associated with MASLD and hepatic fibrosis, suggesting a potential association of spleen volume with the severity of MASLD [14]. Other authors, proposed the longitudinal diameter of the spleen as a non-invasive index for the differentiation of hepatic steatosis from metabolic dysfunction-associated steatohepatitis (MASH) [15], which, however, needs validation by independent cohorts. All the above emphasize the complex pathogenesis of MASLD and its interaction with glucose dysmetabolism, dyslipidemia, and inflammation, whose understanding may be translated in more and better therapeutic options in the future.

Sodium–glucose co-transporter 2 inhibitors (SGLT-2i), initially approved as anti-diabetic medications, have gained growing interest as potential treatment for MASLD, due to their pleiotropic pharmacological actions [16]. SGLT-2i inhibit renal glucose reabsorption, resulting in glycosuria, thus showing a glucose regulating effect, but they have also shown beneficial cardiovascular and renal effects [17]. It has been supported that empagliflozin, an SGLT-2i, may modulate lipid metabolism, and reduce inflammation [18,19], which may both beneficially affect MASLD. More specifically, empagliflozin was shown to reduce lipogenesis and the accumulation of triacylglycerol and lipotoxic intermediates in the liver [19]. Furthermore, empagliflozin was supported to mitigate hepatic inflammation, partly through decreasing the expression of inflammatory cytokines, as well as shifting macrophage polarization to M2 phenotype [18,19].

In this context, the use of suitable preclinical animal models is crucial for more comprehensive understanding of the metabolic pathways underlying the development and progression of MASLD, which is essential for identifying specific therapeutic targets [20]. Towards this aim, a mouse model fed on a fast food diet (FFD), i.e., a diet high in fat, cholesterol, glucose and fructose, a monosaccharide highly prevalent in numerous processed foods that is recognized as a significant contributor to the pathogenesis of MASLD [21], has been supported to mirror human MASLD; this mouse model is known as the FFD model [22,23,24].

Furthermore, lipidomics, the large-scale study of lipids through high-throughput analytical tools, provides critical insights into the lipid alterations in MASLD, thereby elucidating parts of its pathophysiology [25]. This comprehensive approach may focus on both hepatic and circulating lipid profile, leading to the identification of specific lipid alterations that may be linked to the development and progression of MASLD [26], thus possibly leading to the development of non-invasive diagnostic tools [27,28]. Moreover, lipidomics may help to the identification of novel therapeutic targets, which is important for MASLD, for which limited pharmacological interventions exist [26,29].

In our previous work, we investigated the 6-month effect of FFD, with and without empagliflozin treatment, on morphological, biochemical and histological features, as well as on the expression of hepatic genes related to MASLD in mice fed on a FFD [30]. More specifically, when empagliflozin was initiated together with the FFD, i.e., before FFD induces obesity and MASLD, similar weight gain, hepatic steatosis and inflammation and hepatic mRNA expression of key genes of MASLD were observed in mice fed on FFD with and without empagliflozin [30].

In view of the above considerations, the main aim of this study was to investigate whether a 6-month administration of empagliflozin can modulate serum and hepatic lipidomic profiles in a mouse model of MASLD induced by FFD, compared with FFD-fed mice without empagliflozin and control mice fed on a chow diet.

## 2. Results

### 2.1. Animal Study

The comparative morphological and histological data between groups at the endpoint were previously reported [30]. Mean weight gain at the endpoint in the EMPA, FFD and CD groups was 12.6 g, 13.2 g and 7.0 g, respectively, without being statistically significant between groups. After 25 weeks of treatment, histological analysis revealed no significant differences in steatosis grade, lobular inflammation, hepatocellular ballooning, NAFLD activity score (NAS) or fibrosis between the EMPA and FFD groups [30]. However, steatosis and lobular inflammation were more prominent in the EMPA compared with the CD group (*p* = 0.02 and *p* = 0.008, respectively), and hepatocellular ballooning was more prominent in the FFD compared with the CD group (*p* = 0.002). The NAS was significantly greater in the EMPA and FFD compared with the CD group (*p* = 0.002 and *p* = 0.006, respectively). Fibrosis showed a statistically significant trend among the groups, although no significant difference was shown in pairwise comparisons after Bonferroni correction.

### 2.2. Lipid Profiling

In serum, 2860 ion signals in the positive ionization mode and 1920 in negative ionization mode were detected, and in hepatic tissue, 4145 ion signals in the positive ionization mode and 1972 in the negative ionization mode were detected after quality control filtering. These datasets were subjected to analysis using multivariate statistical methods to uncover differently expressed lipidomic phenotype across different groups. The PCA score plots, which include all samples and QC samples, indicated satisfactory analytical precision, as QCs were clustered (Figure S1).

**Figure 1:**
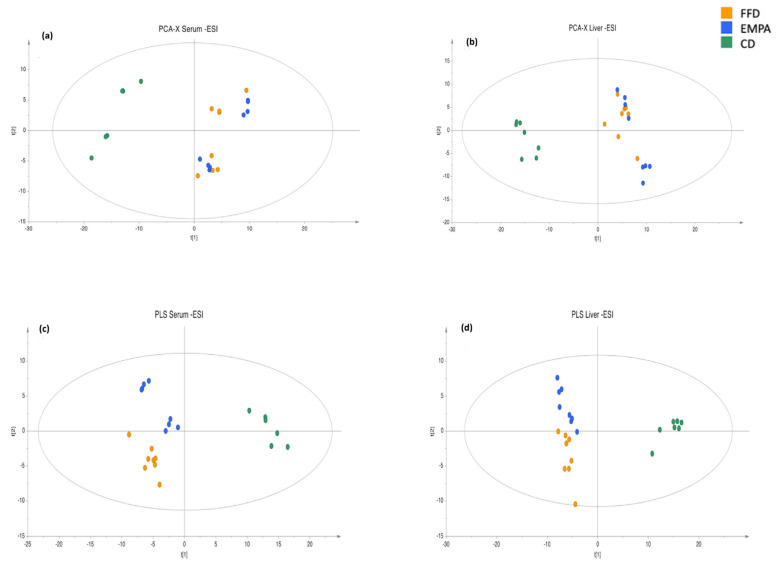
PCA and PLS score plots from the three groups of this study (FFD, EMPA, and CD) in the negative ionization mode. Color represents the group in which each mouse belongs. PCA score plots of (**a**) serum and (**b**) hepatic tissue. The FFD and EMPA groups were discriminated from the CD group. R^2^X = 0.478, Q^2^ = 0.356 for the serum and R^2^X = 0.564, Q^2^ = 0.394 for the liver. PLS score plots of (**c**) serum and (**d**) hepatic tissue. Using supervised models, a clear separation was observed at the plots between FFD vs. CD and EMPA vs. CD, but there was also a definite separation between the FFD and EMPA groups in the serum samples. R^2^X = 0.560, R^2^Y = 0.998, Q^2^ = 0.916, and CV ANOVA = 7.17 × 10^−4^ for the serum and R^2^X = 0.587, R^2^Y = 0.992, Q^2^ = 0.853, and CV ANOVA = 3.85 × 10^−3^ for the liver. Abbreviations: CD, chow diet; CV ANOVA, cross-validated analysis of variance; EMPA, empagliflozin; ESI, electrospray ionization; FFD, fast food diet; PCA, principal components analysis; PLS, partial least squares.

Based on the projection of the serum samples in the PCA models, a clear differentiation of the two FFD groups (FFD and EMPA) from the CD group was observed, irrespective of the treatment parameter (Figure 1a–d and Figure S2). Thus, serum and hepatic lipidomic profiles of the EMPA group showed closer similarity to those of the FFD group than to those of the CD group at the endpoint (25th week).

**Figure 2:**
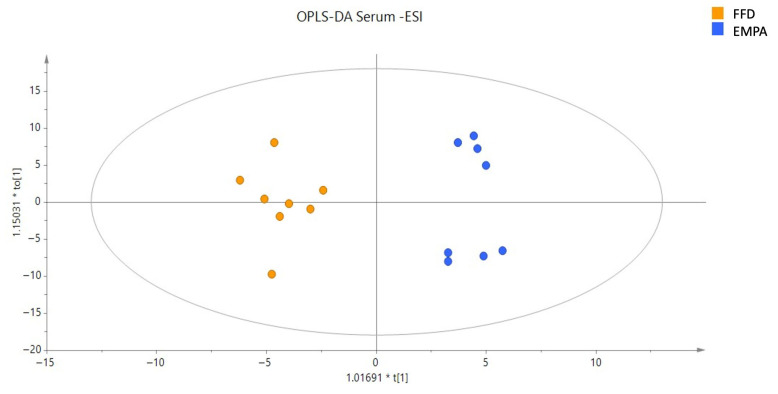
OPLS-DA score plot of serum samples projection based on negative ionization mode analysis data between the FFD and EMPA groups. A definite separation between the FFD and EMPA groups was observed: R^2^X = 0.334, R^2^Y = 0.951, Q^2^ = 0.619, and CV ANOVA = 2.00 × 10^−2^. Abbreviations: EMPA, empagliflozin; ESI, electrospray ionization; FFD, fast food diet; OPLS-DA, orthogonal partial least squares-discriminant analysis.

Following this initial discrimination observed in PCA plots, supervised PLS models were constructed, providing a distinct discrimination between the FFD and EMPA groups in serum (Figure 1c), but not in hepatic samples (Figure 1d), in the negative ionization mode. No discrimination between the FFD and EMPA groups were observed in the positive ionization mode (Figure S2). Subsequently, the FFD group was compared with the EMPA group using OPLS-DA. Specifically, in the positive ionization mode, there was no discrimination between FFD and EMPA in both serum and hepatic samples. On the contrary, separation between the FFD and EMPA groups was observed in serum in the negative ionization mode (Table S1, Figure 2), whereas no separation was observed in hepatic samples in the negative ionization mode.

Because FFD and EMPA mice were discriminated only in the negative ionization mode, we proceeded with further analysis and identification of lipid species on the negative ionization mode. Additionally, since the FFD and EMPA groups were distinct from the CD group in both serum and hepatic tissue samples, a comparison of the two groups on FFD together (EMPA and FFD groups) vs. controls (CD group) was performed to investigate the impact of FFD on the lipidomics (Figure S3, Table S1). The quality metrics of the constructed models were shown in Table S1. The detailed annotations of the identified lipid species in the serum and hepatic samples that were statistically significant between the groups of this study are listed in Table S2.

### 2.3. Lipid Profile in Serum Samples

**Figure 3:**
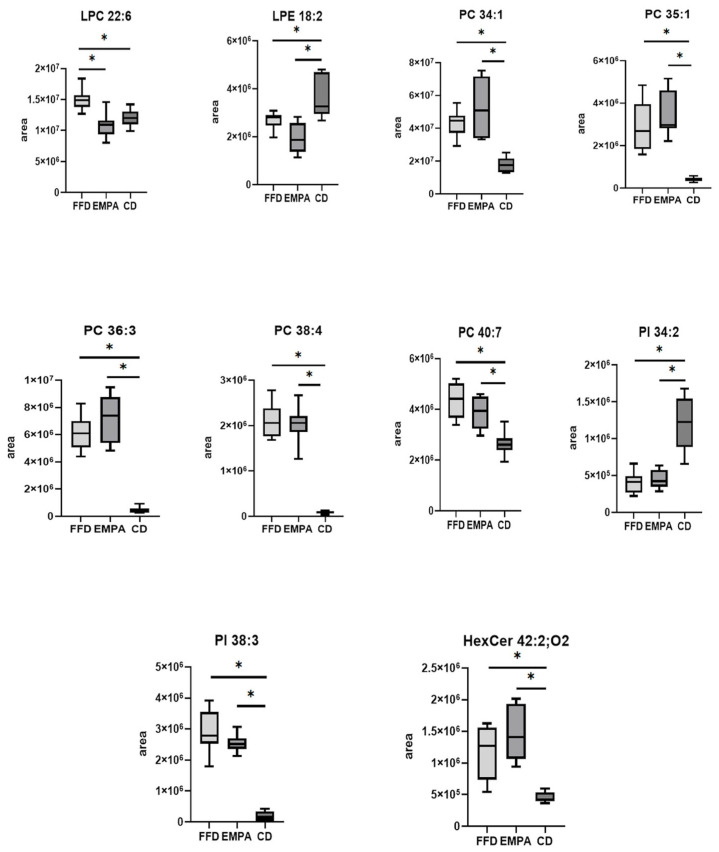
Box plots of identified serum lipid species that were statistically different across the groups. Boxes represent the values between 25% and 75% quartiles. Error bars represent minimum and maximum values. Υ-axis represents the peak areas of lipids displayed on the box plot and this value was derived by the extracted ion chromatogram of the specific lipid. * Indicates *p*-values < 0.05 after Bonferroni correction. Abbreviations: CD, chow diet group; EMPA, empagliflozin group; FFD, fast food diet group; HexCer, hexosylceramide; LPC, lysophosphatidylcholine; LPE, lysophosphatidylethanolamine; PC, phosphatidylcholine; PI, phosphatidylinositol.

Regarding serum samples, the comparison between the FFD and EMPA groups led to the identification of 24 significantly different ions in the negative ionization mode; of them, only 1 was identified, i.e., lysophosphatidylcholine (LPC) 22:6, which was higher in FFD than in the EMPA group (*p* < 0.001, fold change 1.4, Table S2). A larger number of ions were significantly different when comparing the FFD or EMPA group with the CD group. More specifically, 155 ions were identified when comparing the FFD with the CD group, and 150 ions when comparing the EMPA with the CD group; notably, 144 ions were common in these 2 pairs of comparisons. Within them, we identified eight glycerophospholipids and one sphingolipid (Table S2). It was observed that most of these lipid species were higher in the FFD or EMPA compared with the CD group (e.g., PC 34:1, both *p* < 0.001, fold change 2.4 in FFD vs. CD and fold change 2.9 in EMPA vs. CD; PI 38:3, both *p* < 0.001, fold change 17.6 in FFD vs. CD and fold change 15.8 in EMPA vs. CD), apart from lysophosphatidylethanolamine (LPE) 18:2 (both *p* < 0.001 fold change 0.7 and 0.5, respectively) and phosphatidylinositol (PI) 34:2 (both *p* < 0.001, both fold change 0.1), which were lower in the FFD or EMPA compared with the CD group (Figure 3). Regarding the serum lipids per class, LPC, LPE and PI provided a significant trend between groups (at the level of *p* < 0.001; Table S3).

### 2.4. Lipid Profile in Hepatic Tissue Samples

**Figure 4:**
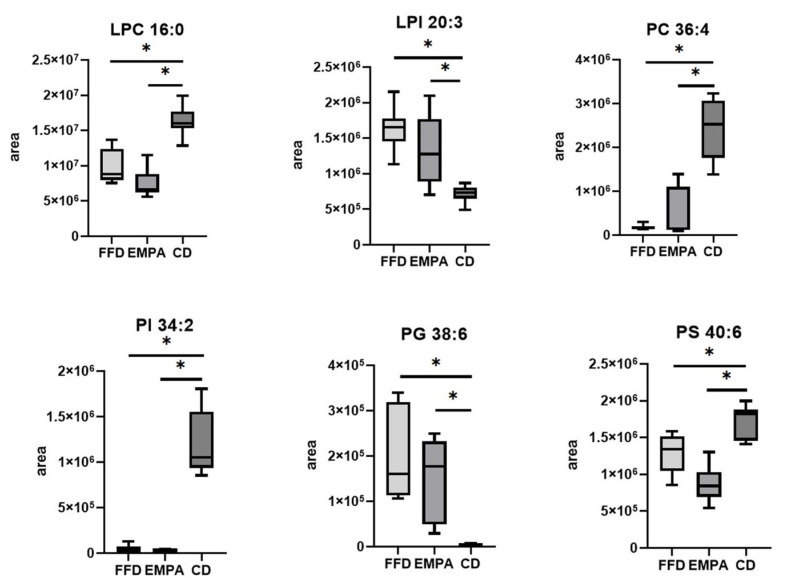
Box plots of identified hepatic lipid species that were statistically different across the groups. Lipids with lowest coefficient of variation are presented. Boxes represent the values between 25% and 75% quartiles. Error bars represent minimum and maximum values. Υ-axis represents the peak areas of lipids displayed on the box plot and this value was derived by the extracted ion chromatogram of the specific lipid. * Indicates *p*-values < 0.05 after Bonferroni correction. Abbreviations: CD, chow diet group; EMPA, empagliflozin group; FFD, fast food diet group; LPC, lysophosphatidylcholine; LPI, lysophosphatidylinositol; PC, phosphatidylcholine; PG, phosphatidylglycerol; PI, phosphatidylinositol; PS, phosphatidylserine.

Although there was not a clear discrimination in either the PCA-X or the PLS plots, differences in 21 ions between the FFD and EMPA groups were shown in the univariate analysis, enabling the identification of the fatty acid (FA) 16:0 (palmitic acid) (*p* < 0.001). Regarding the comparisons between the FFD and CD groups, or between the EMPA and CD groups, 236 and 247, respectively, ions were shown to be significantly different; notably, 236 ions were common in these 2 pairs of comparisons. Within them, we identified 29 glycerophospholipids and 1 sphingolipid (Table S2). Specifically, LPC (e.g., LPC 16:0, both *p* < 0.001, fold change 0.6 in FFD vs. CD and fold change 0.5 in EMPA vs. CD), LPE 18:2 (both *p* < 0.001, fold change 0.6 in FFD vs. CD and fold change 0.5 in EMPA vs. CD), phosphatidylethanolamine (PE) (e.g., PE 34:3, both *p* < 0.001, fold change 0.3 in FFD vs. CD and fold change 0.4 in EMPA vs. CD) and phosphatidylserine (PS) 40:6 (both *p* < 0.001, fold change 0.7 in FFD vs. CD and fold change 0.5 in EMPA vs. CD), were lower in the FFD and EMPA compared with the CD group, while lysophosphatidylinositol (LPI) 20:3 (both *p* < 0.001, fold change 5.0 in FFD vs. CD and fold change 4.2 in EMPA vs. CD), was lower in the CD group. As far as the phosphatidylcholine (PC), PI and phosphatidylglycerol (PG) are concerned, some were higher (e.g., PC 34:1, both *p* < 0.001, fold change 1.7 in FFD vs. CD and fold change 1.6 in EMPA vs. CD), whereas others lower (e.g., PC 32:0, both *p* < 0.001, fold change 0.6 in FFD vs. CD and fold change 0.5 in EMPA vs. CD) in the FFD and EMPA compared with the CD group. Noteworthy, similar patterns were shown for these lipid species in the EMPA and FFD groups, i.e., when they were higher in the FFD compared with the CD group, they were also higher in the EMPA compared with the CD group, and vice versa. As for the ceramide Cer 42:2;O2 it was lower in the FFD and EMPA compared with the CD group (both *p* < 0.001, both fold change 0.7). Identified hepatic lipid species with the lowest coefficient of variation that were different across groups are depicted in Figure 4.

Regarding the hepatic lipids per class, LPC, LPE, LPI, PE and PI provided a significant trend between groups (at the level of *p* < 0.001; Table S3).

### 2.5. Differences Between Male and Female Mice in Serum and Hepatic Samples

**Figure 5:**
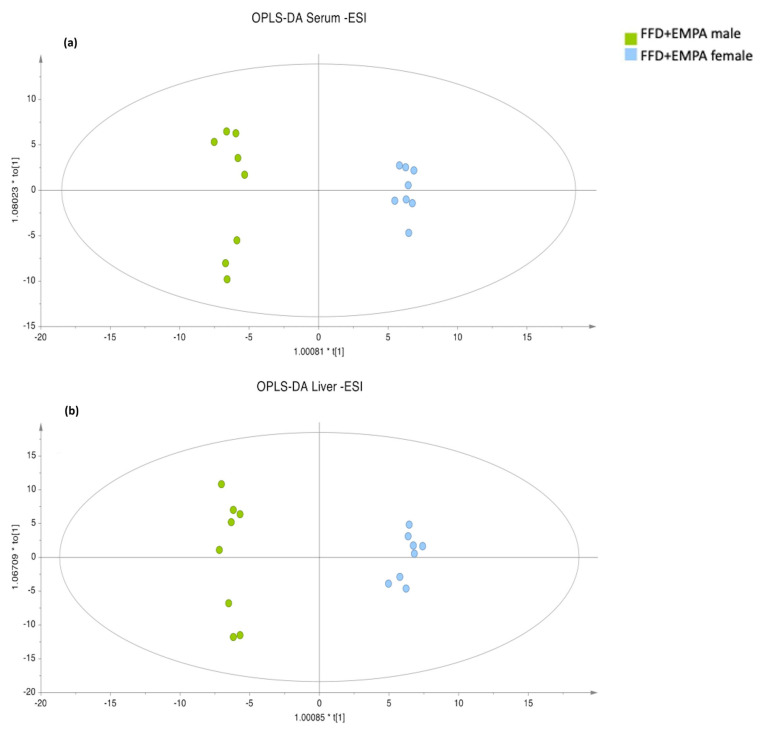
OPLS-DA score plots of mice (**a**) serum and (**b**) hepatic tissue projection from the FFD/EMPA groups between male and female mice based on the negative ionization mode. Discrimination was observed between male and female mice in both serum and hepatic tissue. R^2^X = 0.363, R^2^Y = 0.993, Q^2^ = 0.924, and CV ANOVA = 5.11 × 10^−6^ for the serum and R^2^X = 0.408, R^2^Y = 0.991, Q^2^ = 0.899, and CV ANOVA = 2.39 × 10^−5^ for the liver. Abbreviations: CV ANOVA, cross-validated analysis of variance; EMPA, empagliflozin; ESI, electrospray ionization; FFD, fast food diet; OPLS-DA, orthogonal partial least squares-discriminant analysis.

Next, we investigated for potential sex differences across the FFD, EMPA, and CD groups. Regarding serum samples, there was discrimination between male and female mice in the FFD and EMPA groups in both ionization modes (Table S1). On the contrary, no discrimination was observed between male and female mice in either the positive or negative ionization modes in the CD group. Regarding hepatic samples, there was discrimination between male and female mice in both the positive and negative ionization modes in the EMPA group, as well as in positive, but not the negative mode in the FFD group. On the contrary, no discrimination was observed between male and female mice in hepatic samples in both the ionization modes in the CD group. Such a finding may imply that the impact of FFD may differ between the two sexes. Subsequently, FFD and EMPA male mice (i.e., all male mice fed on FFD) were compared with FFD and EMPA female mice (i.e., all female mice fed on FFD): significantly different ions between these groups were observed in both the positive and negative ionization modes in serum and hepatic tissue samples (Figure 5a,b, Table S1). More specifically, in the serum, there were 96 significantly different ions between FFD/EMPA male and FFD/EMPA female mice, of which 14 were identified, mainly glycerophospholipids (*p* < 0.001 for all lipids). In the liver, there were 67 significantly different ions, of which 5 were identified, mainly sphingolipids (*p* < 0.001 for all lipids, Table S2). There were five common ions in serum and hepatic samples in these comparisons, but no lipid species could be identified among them.

### 2.6. Serum Versus Hepatic Tissue Samples Analysis

Of the 144 significantly different common ions in the comparisons between FFD vs. CD, and EMPA vs. CD in the serum, and the 236 significantly different common ions in the comparisons between FFD vs. CD, and EMPA vs. CD in the liver, 30 were common in both serum and liver. Of these, we identified seven glycerophospholipids (Table S2). More specifically, LPE 18:2 and PI 34:2 were lower in the FFD and EMPA compared with the CD group in both serum and liver, whereas PC 34:1, PC 35:1, PC 36:3, PC 38:4 and PI 38:3 were higher in the FFD and EMPA compared with the CD group in both serum and liver (Table S2).

## 3. Discussion

This was the first study evaluating the effects of a 6-month empagliflozin administration, an SGLT-2i, on serum and hepatic lipidomics in a mouse model of FFD. When considering the results from the untargeted lipidomic analysis, differences in lipidomes were observed mainly in the comparison between the EMPA and CD groups, as well as between the FFD and CD groups, whereas differences between the EMPA and FFD groups were limited. This may imply that the diet itself has a more substantial effect on serum and hepatic lipidomic profile than empagliflozin treatment. These findings are consistent with the lack of significant differences observed in body and hepatic weight, as well as in histological findings between the EMPA and FFD groups, after a 6-month treatment with empagliflozin, as shown to our previous study [30]; in other words, largely similar lipidomic profiles were in line with similar weight and liver gains and similar histological findings in the EMPA and FFD groups, after a 6-month empagliflozin treatment. These findings align with the understanding that diet is a major contributor to the development of MASLD [31], but they should be interpreted in the light of the concomitant initiation of empagliflozin and FFD in this study, i.e., evaluating a preventive rather a therapeutic role of empagliflozin. The main effect of empagliflozin on lipidomics was that observed on LPC 22:6, which was lower in EMPA compared with the FFD group. LPCs, also called lysolecithins, are monoacylglycerophosphocholines, a subclass of lipid species produced by the cleavage of PC, and/or by the transfer of FAs to free cholesterol [32,33]. LPCs are considered to negatively affect the liver [34,35]: they can induce endoplasmic reticulum (ER) stress, disrupt mitochondrial function, and promote apoptosis [36], all being pathogenetically associated with MASLD [4]. Moreover, LPCs can stimulate the generation of extracellular vesicles from the hepatocytes, which activate inflammatory processes in the liver [37].

More differences were observed in serum and hepatic lipidomics in the comparisons of FFD or EMPA with the CD group rather than the comparison between the EMPA and FFD groups (Figure 1 and Figure S2). It is noteworthy that, although most hepatic lipids are stored as triglycerides, a variety of other lipid metabolites, including phospholipids and ceramides, play important pathophysiologic role and have been shown to be accumulated in MASLD [38]. For example, glycerophospholipids and sphingolipids play a role in the structure of membranes or in cell signaling [38,39]. In patients and mice with MASH, previously known as nonalcoholic steatohepatitis (NASH), the membrane integrity was compromised, resulting in the release of its lipids into the hepatic parenchyma [40].

More specifically, lower concentrations of some LPCs in the liver of EMPA/FFD compared with the CD group were observed, which is in accordance with a clinical study of 679 patients with MASLD [41]. LPCs are abundant in high-density lipoprotein-cholesterol (HDL-C), and their reduction seems to be consistent with the inverse association between HDL-C and hepatic steatosis [42,43]. We also indicated lower serum and hepatic concentrations of LPE (18:2) in EMPA/FFD vs. controls, in line with another clinical study with 69 patients with MASLD showing lower serum LPE in patients than controls [44]. Additionally, serum PCs were higher in the EMPA/FFD compared with the control group, which are not all consistent with the finding from the liver, in which some PCs (e.g., PC 34:1, PC 35:1, PC 35:2, PC 36:3, PC 38:4) were higher, while others (e.g., PC 32:0, PC 34:3, PC 36:4, PC 38:6, PC 42:10) lower in the EMPA/FFD than the CD group (Table S2).

Furthermore, some hepatic PEs were lower in the EMPA/FFD compared with the CD group. PCs and PEs are the primary phospholipids found in mammalian cell membranes and their balance on the surface of lipid droplets within the hepatocyte is essential for the dynamics of the membranes [45,46]. PCs and PEs may also cross-talk, since PC synthesis can be maintained through the methylation of PE [47]. As the methylation of PE produces more PC, the PE content is reduced, but the excess PC leads to triglyceride synthesis within the hepatocyte, i.e., steatosis [48,49]. In light of the above, the association of PCs and PEs with MASLD, particularly the PC-to-PE ratio, has been supported to be critical for various tissues, including the liver, in which both low and high PC-to-PE ratio were associated with the development of MASLD and the severity of the disease [46]. In other words, adequate PC is crucial for the production and exportation of very low density lipoprotein (VLDL) from the hepatocyte, but excessive PC, e.g., through the methylation of PE, may lead to excessive VLDL within the hepatocyte that cannot be all exported. Similar to the findings reported in our study, another study of metabolic pathways associated with fat accumulation in bearded dragons pointed to relevant changes in the hepatic lipidome as hepatic fat content increases [50]. Specifically, glycerolipids tend to increase, while phospholipids, sphingolipids, and sterol lipids tend to decrease. The lipid species that were most strongly associated with decreasing hepatic fat content were PEs, one PC and one sphingomyelin [50].

Moreover, the concentrations of some PI were increased (e.g., PI 38:3) or reduced (e.g., PI 34:2) in the serum and in hepatic samples of EMPA/FFD mice compared with CD mice. Limited relevant data also showed opposite trends of different PI concentrations in patients with MASLD compared with controls [40,51]. PI were generally implicated in promoting the transfer of cholesterol to HDL-C, while impeding its transport and storage in low-density lipoprotein-cholesterol (LDL-C) [52]. Of course, the results of this study warrant mechanistic studies to elucidate the specific role of different PI, which is largely unknown.

Interestingly, hepatic PS 40:6 levels were lower in EMPA/FFD compared with the CD group in this study. Similarly, Chiappini et al. reported lower total hepatic PS in patients with MASH compared with controls, but not significantly different PS between patients with hepatic steatosis and controls [40]. Despite the reduced concentrations of plasma PS, their precise pathophysiologic role in MASLD remains unclear; although it remains to be shown, PS could potentially affect the structure and functionality of lipoproteins, including HDL-C [53].

Additionally, most hepatic PGs were higher (e.g., PG 38:4, PG 38:6 and PG 38:7) in EMPA/FFD compared with the CD group. This is consistent with other studies that showed an abundance of PGs in hepatic areas of steatosis and in the circulation of patients with MASH compared with those without MASH [54,55]. PGs are primarily found in bacterial membranes, whereas the membranes of eukaryotic cells predominantly contain PCs [56]. Notably, altered gut microbiota influences intestinal permeability through the production of toxic bacterial products, thus increasing bacterial translocation and inflammatory mediators, which impacts the liver via the gut–liver axis, thereby promoting the progression of MASLD [57]. Therefore, increased concentrations of PGs in MASLD may be associated with altered gut microbiota and could indicate the effect of microbial products, which, however, remains to be definitely shown.

As for the use of NITs, another study which compared patients with MASLD and obesity with healthy controls, supported that the majority of the highly correlated lipid species between plasma and liver belonged to phospholipid classes, including PC, LPC, PE, PI and SM [58]. In another study of patients with MASLD, PC 38:4 was similarly affected in the liver and serum, as in our study [59]. However, the specific influence of PC 38:4 on the progression or regression of MASLD is uncertain based on the available evidence [60,61]. Another study of mice fed on methionine choline-deficient diet identified 37 significant lipid species in the serum and 38 significant lipid species in the liver compared with controls; 4 of them (cholesterol, myristic acid, palmitoleic acid and docosapentanoic acid) were similarly different in the serum and the liver [62]. Furthermore, more lyso-species were identified in the liver compared with serum in another study with MASLD patients compared with controls [63]. However, no definite conclusion could be made when comparing the above mentioned studies. Different models/populations, diets, interventions and study durations practically render the results of these studies hard to compare.

Concerning the differences between male and female mice, elevated concentrations of some sphingomyelins, which are the most common subgroup of sphingolipids, were observed in individuals with high HDL-C concentrations [64], which are also lower in males than females. It is important to note that most studies with mouse models of MASLD include only male mice, thus there is a general underrepresentation of female mice in the literature, which is highlighted, since the results of male could not always be extrapolated to female mice [65,66,67]. The observed limited impact of empagliflozin on lipidomics in this study may be, at least partly, attributed to the timing of initiation of empagliflozin. Administering empagliflozin together with the initiation of FFD, i.e., before the establishment of glucose or lipid dysmetabolism or hepatic steatosis, may have restricted the potential for detectable modulation of lipidomics. The mechanisms of action of empagliflozin, including glycosuria, stimulation of fatty acid oxidation, and decrease in hepatic lipogenesis, may be less effective in the absence of relevant metabolic aberrations [68]. In other words, empagliflozin may offer limited benefits as a preventive measure against FFD, which may be different from the effect of empagliflozin as a therapeutic measure, i.e., if it is administered after the development of obesity, diabetes, MASLD and dyslipidemia by the FFD. In this regard, when empagliflozin was administered for 12 weeks after the development of fibrotic MASLD in mice previously being for 36 weeks on the amylin liver NASH diet (AMLN), mice on AMLN/empagliflozin provided a distinct hepatic lipidomic cluster in PLS-DA compared with mice on AMLN/vehicle (controls), mainly owing to some lactosylceramides (that were lower in empagliflozin than the control group) and polyunsaturated triglycerides (that were higher in empagliflozin than the control group) [69]. However, similarly to our findings, empagliflozin did not lead to weight loss or histological improvement compared with the control group, apart from a marginal decrease in NAS, in this study [69]. It should be also underlined that AMLN diet differs from the FFD, most notably in its substantially higher cholesterol content (2% vs. 0.2%, respectively) [22]. There are also some more studies suggesting that SGLT-2i may have therapeutic efficacy in animal models or patients with pre-existing metabolic or hepatic pathology [69,70,71]. In light of the above, a distinct preventive and therapeutic effect of empagliflozin may be supported, which, however, needs the validation of other studies.

The originality is a strength of this study, which investigated the effect of empagliflozin, a commonly prescribed anti-diabetic medication, on the lipidomic profile of mice fed on FFD. A key strength is the simultaneous evaluation of lipidomics in both serum and liver, allowing a comprehensive comparative assessment of serum and liver-specific effects. Furthermore, the concomitant initiation of empagliflozin with FFD was another advantage, targeting to evaluate the preventive, rather the therapeutic, effects of empagliflozin against FFD. However, this study has certain limitations: (a) The FFD model did not reproduce all phenotypes of MASLD, as elsewhere discussed [24]. (b) The sample size per group (*n* = 8) may have limited the ability to detect subtle or moderate lipidomic differences, especially between the EMPA and FFD groups; however, power analysis is hardly feasible in an untargeted lipidomic analysis. This limitation is further emphasized in the comparisons between subgroups stratified by sex (four mice per group), so these findings should be interpreted with caution, as they may be underpowered. (c) A specific statistical test for False Discovery Rate correction was not applied to comparisons between groups; however, in order to limit the risk of false positive results, we had chosen to set the *p*-value to a more rigorous cut-off than usually (*p* < 0.001). (d) The results of total lipid quantification per class of lipids (Table S3) should be interpreted with caution, because not all lipid species of a class follow the same pattern; in this regard, some lipid species maybe increased, other maybe decreased and other maybe similar in the comparisons between groups within a lipid class (Table S2). (e) Studies of longer duration may reveal more differences in lipidomics, specifically for the comparison between EMPA and FFD.

In conclusion, empagliflozin had limited effects on the overall lipidomic profile in mice fed a FFD, although specific changes, such as lower LPC (22:6) levels, were observed between the EMPA and FFD groups. However, both the FFD and EMPA groups showed distinct lipidomic profile compared with the CD group, implying the effect of diet on lipidomic profile. Several lipid species were consistently altered in both serum and liver; this holds promise for targeted studies evaluating the specific lipid species in the setting of the development of new NITs for MASLD. Mechanistic studies are also needed to investigate the role of specific lipid species in the pathophysiology of MASLD.

## 4. Materials and Methods

### 4.1. Animal Study

This is a follow-up study of our previous work, which had been focused on the comparative difference of biochemical, genetic and histological parameters between groups [30]. C57BL/6J mice (male and female) were purchased by and bred in the animal facility of the Laboratory of Anatomy, Histology & Embryology, School of Veterinary Medicine, Aristotle University of Thessaloniki, Greece, at the age of eight to nine weeks. Mice were from the same strain (inbred strain; same genetic background) and were bred in the colony according to the approved breeding scheme by the designated veterinarian of the facility. Each group consisted of 8 mice, 4 male and 4 female. Mice were housed in a pathogen-free, controlled environment (12/12 h light/dark cycle, 20 ± 2 °C temperature, 60% humidity). At the baseline, the animals were randomly assigned into three groups, stratified by sex. One group (empagliflozin group, EMPA) received sterilized FFD (TD.88137, ssniff Spezialdiäten GmbH, Soest, Germany) and empagliflozin embedded into food pellets (10 mg/kg/day, as previously described [72,73]) with fructose (23.1 g/L) and glucose (18.9 g/L) (Sigma-Aldrich Chemie GmbH, Schnelldorf, Germany) in the drinking water; another group (FFD group) received the same diet (FFD with fructose and glucose), but without empagliflozin; a third group (chow diet group, CD) received sterilized CD (V1534-703, ssniff Spezialdiäten GmbH, Soest, Germany) without fructose and glucose, as previously reported [24]. The particular nutritional components of each diet are summarized in Table S4. The animals had ad libitum access to food and water for six months (25 weeks). Four mice of the same sex, fed on the same diet were housed in each cage, which was appropriately cleaned once per week. At week 25, mice were euthanatized after being anesthetized with isoflurane inhalation (Iso-Vet, Biovet, Thessaloniki, Greece). Weight was measured and non-fasting blood samples were collected via cardiac puncture before euthanasia. Blood samples were incubated for 60 min at room temperature and centrifuged at 13,000× *g* for 15 min at 8 °C. Serum was collected and stored at −80 °C. Hepatic tissue samples were also collected; a part of hepatic tissue was immediately transferred into 10% buffered formalin and embedded into paraffin, whereas another part was stored in tubes in liquid nitrogen and subsequently frozen at −80 °C, as previously reported [24]. For consistency, liver tissue was collected from the same anatomical regions in all animals: the right lobe was used for histological analysis, and the left lobe was used for lipidomic analysis.

All animal procedures were carried out in accordance with the relevant National and European Union regulations and were approved by the General Directorate of Agriculture, Economy and Veterinary Medicine of the region of Central Macedonia, Greece, as well as by the Bioethics Committee, School of Medicine, Aristotle University of Thessaloniki, Greece (approval number 5347, approval date: 23 February 2021).

### 4.2. Samples Preparation for Lipidomic Analysis

For the untargeted lipidomic analysis in serum, 50 µL of serum samples were thawed on ice for 30 min. Lipids extraction was performed by adding 375 µL methanol (MeOH, CHEM-LAB NV, Zedelgem, Belgium) and 1250 µL of methyl-tert-butyl-ether (MTBE; ≥99%; CHEM-LAB NV, Zedelgem, Belgium) in the samples, followed by vortexing and shaking for 1 h at room temperature. After the addition of 375 µL deionized H_2_O (ddH_2_O; Millipore Bedford, Bedford, MA, USA), the agitation was repeated for 10 min at ambient temperature. Then, the extracts were centrifuged at 11,180× *g* for 10 min at 4 °C. The organic phase layer was transferred to 2 mL Eppendorf tubes and evaporated under vacuum (SpeedVac, Eppendorf Austria GmbH, Vienna, Austria). Finally, the extracts residues were reconstituted in 1200 μL of isopropanol (IPA; Fisher Scientific International Inc., Hampton, NH, USA).

Regarding lipidomic analysis in hepatic tissues, the thawed hepatic tissues were transferred into 2.0 mL Eppendorf tubes containing 1.0 mm ceramic beads, after weighing. The organic solvent mixture of MTBE-MeOH (CHEM-LAB NV, Zedelgem, Belgium) 3:1 (*v*/*v*) was added to the tissue for lipid extraction, proportionally to the weight and up to 1200 μL for the maximum weight. The tissues were homogenized using a Bead mill Homogenizer (Bead Ruptor Elite, Omni International, Kennesaw, GA, USA), in 4 cycles (30 s duration each and at 6.00 m/s speed). After homogenization, the mixtures were centrifuged at 16,770× *g* for 30 min at 4 °C and then 300 µL of the supernatant were transferred to a 1.5 mL Eppendorf tube, followed by evaporation to dryness. The dried extracts were reconstituted in 450 μL of H_2_O–acetonitrile (ACN)–IPA in a 1:1:3 (*v*/*v*/*v*) ratio before the analysis.

Additionally pooled samples (QC Sample) were prepared for both matrices by pooling equal volumes of all extracts for data quality assessment. In addition, specific group QC samples were prepared for each of the three groups of this study. Furthermore, diluted QCs (1:2, 1:4, 1:6, 1:8) were prepared to evaluate the dilution integrity of the detected signals.

### 4.3. Samples Analysis

The analytical conditions applied were previously described [25]. An Elute Ultra High-Performance Liquid Chromatography system, (UHPLC; Elute system, Bruker, Bremen, Germany) equipped with an Elute autosampler was utilized. Autosampler temperature was set at 8 °C; before and after each injection the needle was washed with IPA/ACN 90:10 (*v*/*v*; 1000 μL), and then with ACN/H_2_O 60/40 (*v*/*v*; 1000 μL). The separation of lipids was performed on an Acquity UPLC CSH C18, 2.1 × 100 mm, 1.7 μm column (Waters Ltd., Elstree, UK) using a pre-column Acquity UPLC CSH C18Van-Guard (Waters Ltd., Elstree, UK). A 20-min gradient elution was employed using a mobile phase A consisting of ACN/H_2_O 60:40 (*v*/*v*), 10 mM ammonium formate (Sigma-Aldrich, Merck, Darmstadt, Germany), and 0.1% formic acid (CHEM-LAB NV, Zedelgem, Belgium), and a mobile phase B consisting of IPA/ACN 90:10 (*v*/*v*) and 0.1% formic acid. A multi-step gradient from 60% to 1% (mobile phase A) was applied over a period of 18 min. The flow rate was set at 0.4 mL/min and column oven temperature at 55 °C. For the analysis of serum and hepatic samples, 5 μL and 8 μL were injected in the positive and negative ionization modes.

High-resolution mass spectrometry (MS) was performed by a time-of-flight (TOF) mass spectrometer (Bruker, Bremen, Germany) for both the positive and negative ionization modes, performing data-dependent acquisition (DDA) for MS/MS analyses. The electrospray ionization (ESI) settings were as follows: capillary voltage ±4.2 kV, dry temperature 200 °C, dry gas 10 L/min and nebulizer gas 2.0 Bar. Auto MS/MS was applied using dynamic MS/MS spectra acquisition with a minimum and maximum spectra rate of 6 and 10 Hz, respectively. In DDA analysis, MS/MS spectrums were retrieved for the 10 most intense ions per scan. The collision energy was set at 20 V for precursor ions with *m*/*z* < 100, 30 V for precursor ions with m/z ranging from 100 to 1000, and 40 V for precursor ions with *m*/*z* ranging from 1000 to 2000. During each analysis, a calibrant (sodium formate, 10 mM) was infused into the MS at a flow rate of 10 µL/h during 0.1–0.3 min [25].

Each matrix was analyzed in a separate analytical batch. The samples were analyzed in a random order, with QC samples analyzed every 5 samples. Prior to the analysis of individual samples, blank samples, procedural blank samples, 4 QCs for system equilibration and diluted QCs were analyzed.

### 4.4. Data Analysis

Data from TOF MS were recalibrated using sodium formate clusters through the vendor’s Data Analysis software (version 5.3, Bruker Bremen, Bremen, Germany) and then converted to mzML format using MSConvert (ProteoWizard 3.0.11567, Palo Alto, CA, USA). An in-house chromatography MS (XCMS) script of various forms of (XCMS) implemented in the R programming (version 3.2.0, R Foundation, Vienna, Austria) was employed to perform retention time alignment and feature grouping prior to chromatographic peak identification. Any features with coefficient of variation (CV) values > 30% in QCs were removed from further analysis. Multivariate statistical analysis was carried out with SIMCA 13.0.3 software package (UMETRICS Umea, Umea, Sweden) [74]. Unsupervised principal component (PCA), partial least squares (PLS) and orthogonal-partial least squares discriminant (OPLS-DA) analysis were applied. The S-plot filter was employed to identify significant features, applying absolute p and p corrected [p(corr)] cut-off values of >|0.05| and >|0.5|, respectively. Parameters related to the quality of the models, including goodness of fit in the X (R^2^X) and Y (R^2^Y) variables, predictability (Q^2^YCV) and *p*-values from cross-validated analysis of variance (CV ANOVA) were calculated from the software. Logarithmic transformation of the data and pareto scaling were used in all multivariate models.

Statistical analysis was performed using R Studio, version 1.4.1717 (R Foundation for statistical computing, Vienna, Austria). The normality of distributions was evaluated with the Shapiro–Wilk test. For normally distributed continuous variables, one-way ANOVA was used, followed by Bonferroni correction, when the *p*-value for trend of ANOVA was statistically significant; for non-normally distributed continuous variables, the Kruskal–Wallis test was used, followed by Dunn’s test to correct for multiple pairwise comparisons, when the *p*-value for trend in the Kruskal–Wallis was statistically significant. The homogeneity of variance was tested with the Levene’s test. The chi-square test was used to compare categorical variables. The *p*-value was set to <0.001 in the comparisons of lipid species, in order to limit the risk of false positive results, given the numerous lipid species that were retrieved. However, this *p*-value was not applied to the pairwise comparisons after Bonferroni correction, to avoid over-correction.

### 4.5. Identification of Lipid Species

Lipid species that were statistical significant between groups were identified using the Lipostar2 (version 2.0.2, Molecular Discovery Ltd., Hertfordshire, UK), integrated in the LIPID MAPS structure database (https://www.lipidmaps.org; multi-institutional supported database) [75]. The raw data were imported and aligned using the default settings. Automatic peak detection was performed with the Savitzky–Golay algorithm, using the following settings: window size of 7, degree to 2, multi-pass iterations of 1, and a minimum signal-to-noise (S/N) ratio of 3. Mass tolerance was set to 10 ppm with a retention time (RT) tolerance of 0.2 min. To focus on features with isotopic patterns and MS/MS spectrums, filters were applied to retain only lipids with isotopic pattern and retain lipids with MS/MS. The lipid identification process was performed with a precursor ion mass tolerance of 5 ppm and a product ion mass tolerance of 20 ppm. The automatic approval applied for structures with a quality rating between 3 and 4 stars. Finally, the intensities of lipids were summed up to provide an indication of changes per class.

## Data Availability

The dataset generated during this study, including the complete data of lipidomics, is available by the corresponding author upon reasonable request.

## Supplementary Materials

The supporting information can be downloaded at: https://www.mdpi.com/article/10.3390/ijms26199273/s1.
