# Supplementary material for: Serum and Liver Lipidome Following Empagliflozin Administration for Six Months in a Fast Food Diet Mouse Model
Source: Int J Mol Sci. 2025 Sep 23;26(19):9273. doi: 10.3390/ijms26199273 (PMC12524644; doi:10.3390/ijms26199273)
Supplement: Supplementary file 1 [file ijms-26-09273-s001.zip › Legends to Supplementary Figures.pdf]

Figure S1. PCA score plots of mice in serum and hepatic tissue of the three groups of the study and QC samples. QC samples are presented with dark red color and are clustered together. (a) Serum samples projection based on positive ionization mode analysis data: $R^2X=0.656$, $Q^2=0.446$. (b) Serum samples based on negative ionization mode data: $R^2X=0.539$, $Q^2=0.407$. (c) Liver tissue samples based on positive ionization mode data: $R^2X=0.679$, $Q^2=0.443$. (d) Liver tissue samples based on negative ionization mode data: $R^2X=0.607$, $Q^2=0.424$. Abbreviations: CD, chow diet; EMPA, empagliflozin; ESI, electrospray ionization; FFD, fast food diet; PCA, principal components analysis; QC, quality control.

Figure S2. PCA and PLS score plots from the three groups of the study (FFD, EMPA, and CD) in positive ionization mode. Colour represents the group in which each mouse belongs. PCA score plots of (a) serum and (b) hepatic tissue. FFD and EMPA groups were discriminated from CD group. $R^2X=0.601$, $Q^2=0.374$ for the serum and $R^2X=0.528$, $Q^2=0.390$ for the liver. PLS score plots of (c) serum and (d) hepatic tissue. Using supervised models, a clear separation is observed at the plots between FFD vs. CD and EMPA vs. CD, but there is not a definite separation between FFD and EMPA group either in serum or in the liver tissue on positive ionization mode data. $R^2X=0.549$, $R^2Y=0.948$, $Q^2=0.760$, CV ANOVA=7.67×10^{-4} for the serum and $R^2X=0.552$, $R^2Y=0.993$, $Q^2=0.818$, CV ANOVA=9.33×10^{-4} for the liver. Abbreviations: CD, chow diet; CV ANOVA, cross-validated analysis of variance; EMPA, empagliflozin; ESI, electrospray ionization; FFD, fast food diet; PCA, principal components analysis; PLS, partial least squares.

Figure S3. OPLS-DA score plots of serum and hepatic tissue in negative ionization mode. There is a definite separation between groups (FFD vs. CD and EMPA vs. CD) in the serum and hepatic tissue. (a) Serum samples projection based on negative ionization mode analysis data between the FFD and CD group: $R^2X=0.523$, $R^2Y=0.993$, $Q^2=0.961$, CV ANOVA=3.43×10^{-6}. (b) Serum samples projection based on negative ionization mode between the EMPA and CD group: $R^2X=0.563$, $R^2Y=0.997$, $Q^2=0.972$, CV ANOVA=7.67×10^{-7}. (c) Hepatic tissue samples projection based on negative ionization mode between FFD and CD group: $R^2X=0.500$, $R^2Y=0.996$, $Q^2=0.975$, CV ANOVA=9.21×10^{-8}. (d) Hepatic tissue samples projection based on negative ionization mode between EMPA and CD group: $R^2X=0.562$, $R^2Y=0.996$, $Q^2=0.980$, CV ANOVA=2.68×10^{-8}. Abbreviations: CD, chow diet; EMPA, empagliflozin; ESI, electrospray ionization; FFD, fast food diet; OPLS-DA, orthogonal partial least squares-discriminant analysis.
