# Supplementary material for: Serum and Liver Lipidome Following Empagliflozin Administration for Six Months in a Fast Food Diet Mouse Model
Source: Int J Mol Sci. 2025 Sep 23;26(19):9273. doi: 10.3390/ijms26199273 (PMC12524644; doi:10.3390/ijms26199273)
Supplement: Supplementary file 1 [file ijms-26-09273-s001.zip › Polyzos_Table S1.pdf]

Table S1. Characteristics of the constructed unsupervised and supervised models.

Models	Type	N	R^2X	R^2Y	Q^2	CV ANOVA
Serum -ESI						
FFD-EMPA-CD-QCs	PCA-X	27	0.539		0.407	
FFD-EMPA-CD	PCA-X	22	0.478		0.356	
FFD-EMPA-CD	PLS	22	0.560	0.998	0.916	7.17×10^{-4}
FFD-CD	OPLS-DA	14	0.523	0.993	0.961	3.43×10^{-6}
EMPA-CD	OPLS-DA	14	0.563	0.997	0.972	7.67×10^{-7}
FFD-EMPA	OPLS-DA	14	0.334	0.951	0.619	2.00×10^{-2}
FFD Male-FFD Female	OPLS-DA	8	0.468	0.999	0.870	3.00×10^{-2}
EMPA Male-EMPA Female	OPLS-DA	8	0.527	0.999	0.944	6.07×10^{-3}
FFD+EMPA Male vs FFD+EMPA Female	OPLS-DA	16	0.363	0.993	0.924	5.11×10^{-6}
Serum +ESI						
FFD-EMPA-CD-QCs	PCA-X	28	0.656		0.446	
FFD-EMPA-CD	PCA-X	23	0.601		0.374	
FFD-EMPA-CD	PLS	23	0.549	0.948	0.760	7.67×10^{-4}
Liver -ESI						
FFD-EMPA-CD-QCs	PCA-X	28	0.607		0.424	
FFD-EMPA-CD	PCA-X	23	0.564		0.394	
FFD-EMPA-CD	PLS	23	0.587	0.992	0.853	3.85×10^{-3}
FFD-CD	OPLS-DA	15	0.500	0.996	0.975	9.21×10^{-8}
EMPA-CD	OPLS-DA	15	0.562	0.996	0.980	2.68×10^{-8}
EMPA Male-EMPA Female	OPLS-DA	8	0.555	0.999	0.934	8.67×10^{-3}
FFD+EMPA Male vs FFD+EMPA Female	OPLS-DA	16	0.408	0.991	0.899	2.39×10^{-5}
Liver +ESI						
FFD-EMPA-CD-QCs	PCA-X	28	0.679		0.443	
FFD-EMPA-CD	PCA-X	23	0.528		0.390	
FFD-EMPA-CD	PLS	23	0.552	0.993	0.818	9.33×10^{-4}

Log transformation and PAR scale were used in all models in serum and liver ± ESI models.

Abbreviations: ; CD, chow diet; EMPA, empagliflozin; ESI, electrospray ionization; FFD, fast food diet; OPLS-DA, orthogonal partial least squares-discriminant analysis; PAR, pareto; PCA, principal components analysis; PLS, partial least squares; QC, quality control.
