# Supplementary material for: Serum and Liver Lipidome Following Empagliflozin Administration for Six Months in a Fast Food Diet Mouse Model
Source: Int J Mol Sci. 2025 Sep 23;26(19):9273. doi: 10.3390/ijms26199273 (PMC12524644; doi:10.3390/ijms26199273)
Supplement: Supplementary file 1 [file ijms-26-09273-s001.zip › Polyzos_Table S2.pdf]

Table S2. Summary of identified lipid species in the serum and hepatic samples being statistically significant between the three groups of the study.

Bulk number	Annotations	Neutral formula	Exact mass	Exact mass adduct (*m/z*)	Monoisotopic mass adduct (*m/z*)	Δ ppm	Rt min	Adduct	p-value for trend [a]	Pairwise comparisons (corrected p-values) [a]		Log_2FC	CV%
FFD vs. EMPA (in the serum)													
LPC 22:6	LPC 22:6	C30H50NO7P	567.3325	612.3241	612.3307	-10.6	1.0	[M+HCOO]-	3.94E-04	3.60E-04		0.47	7.57
Common lipids observed in the comparisons between FFD vs. CD and EMPA vs. CD (in the serum)													
LPE 18:2*	LPE 18:2	C23H44NO7P	477.2855	476.2744	476.2783	-8.1	1.2	[M-H]-	4.26E-04	FFD vs. CD	3.70E-02	-0.44	5.61
										EMPA vs. CD	3.00E-04	-0.89	
PC 34:1*	PC 16:0_18:1	C42H82NO8P	759.5778	804.5685	804.5760	-9.3	7.9	[M+HCOO]-	3.43E-04	FFD vs. CD	5.92E-03	1.27	6.27
										EMPA vs. CD	2.90E-04	1.55	
PC 35:1*	PC 17:0_18:1	C43H84NO8P	773.5935	818.5899	818.5917	-2.2	8.2	[M+HCOO]-	3.10E-05	FFD vs. CD	4.20E-04	2.82	13.5
										EMPA vs. CD	3.30E-05	3.10	
PC 36:3*	PC 18:1_18:2	C44H82NO8P	783.5778	828.5651	828.5760	-13.1	7.0	[M+HCOO]-	1.15E-04	FFD vs. CD	7.40E-04	0.70	8.22
										EMPA vs. CD	1.60E-04	0.78	
	PC 16:0_20:3	C44H82NO8P	783.5778	828.5663	828.5760	-11.7	7.6	[M+HCOO]-	3.63E-08	FFD vs. CD	7.00E-07	3.78	10.3
										EMPA vs. CD	4.70E-08	4.02	
PC 38:4*	PC 18:1_20:3	C46H84NO8P	809.5935	854.5933	854.5917	1.9	7.2-7.8	[M+HCOO]-	8.04E-10	FFD vs. CD	2.60E-09	4.60	21.4
										EMPA vs. CD	4.80E-09	4.55	
PC 40:7	PC 18:1_22:6	C48H82NO8P	831.5778	876.5716	876.5760	-5.1	6.1	[M+HCOO]-	3.33E-04	FFD vs. CD	2.80E-04	0.72	6.87
										EMPA vs. CD	6.11E-03	0.55	
PI 34:2*	PI 34:2	C43H79O13P	834.5258	833.5134	833.5186	-6.3	4.9	[M-H]-	1.09E-11	FFD vs. CD	4.70E-11	-2.83	10.4
										EMPA vs. CD	5.50E-11	-2.75	
PI 38:3*	PI 18:0_20:3	C47H85O13P	888.5728	887.5636	887.5655	-2.2	7.2	[M-H]-	1.96E-10	FFD vs. CD	3.60E-10	4.14	3.31
										EMPA vs. CD	2.80E-09	3.98	
HexCer 42:2;O2	HexCer 42:2;O2	C48H91NO8	809.6745	854.6698	854.6727	-3.4	9.0	[M+HCOO]-	2.88E-07	FFD vs. CD	2.50E-06	2.79	14.6
										EMPA vs. CD	5.20E-07	2.92	
Common lipids observed in the comparisons between FFD vs. CD and EMPA vs. CD (in the liver)													
LPC 16:0	LPC 16:0	C24H50NO7P	495.3325	540.3252	540.3307	-10.2	1.3	[M+HCOO]-	7.22E-07	FFD vs. CD	5.20E-05	-0.72	2.62
										EMPA vs. CD	6.40E-07	-1.11	
LPC 18:2	LPC 18:2	C26H50NO7P	519.3325	564.3261	564.3307	-8.1	1.1	[M+HCOO]-	6.32E-11	FFD vs. CD	2.00E-10	-4.00	4.78
										EMPA vs. CD	7.90E-10	-2.92	
LPC 18:3	LPC 18:3	C26H48NO7P	517.3168	562.3112	562.3150	-6.7	1.0	[M+HCOO]-	6.50E-09	FFD vs. CD	3.00E-08	-3.28	11.1
										EMPA vs. CD	2.60E-08	-3.28	
LPC 20:4	LPC 20:4	C28H50NO7P	543.3325	588.3241	588.3307	-11.2	1.1	[M+HCOO]-	1.25E-06	FFD vs. CD	3.60E-05	-0.97	4.31

										EMPA vs. CD	1.50E-06	-1.38	
LPE 18:2*	LPE 18:2	C23H44NO7P	477.2855	476.2744	476.2783	-8.1	1.2	[M-H]-	5.32E-07	FFD vs. CD	6.10E-06	-1.36	5.18
										EMPA vs. CD	1.10E-06	-1.69	
LPI 20:3	LPI 20:3	C29H51O12P	622.3118	621.2995	621.3045	-8.1	1.0	[M-H]-	3.80E-06	FFD vs. CD	4.30E-06	2.33	2.19
										EMPA vs. CD	1.00E-04	2.06	
PC 32:0	PC 16:0_16:0	C40H80NO8P	733.5622	778.5544	778.5604	-7.7	7.8	[M+HCOO]-	7.02E-05	FFD vs. CD	5.60E-04	-0.74	17.7
										EMPA vs. CD	1.10E-04	-0.90	
PC 34:1*	PC 16:0_18:1	C42H82NO8P	759.5778	804.5678	804.5760	-10.2	7.9	[M+HCOO]-	2.27E-06	FFD vs. CD	5.50E-06	0.74	4.24
										EMPA vs. CD	1.60E-05	0.70	
PC 34:3	PC 16:1_18:2	C42H78NO8P	755.5465	800.5401	800.5447	-5.7	5.3	[M+HCOO]-	2.92E-05	FFD vs. CD	4.90E-05	-1.63	13.7
										EMPA vs. CD	2.50E-04	-1.29	
	PC 16:0_18:3	C42H78NO8P	755.5465	800.5387	800.5447	-7.5	5.8	[M+HCOO]-	1.16E-04	FFD vs. CD	1.10E-04	-0.86	23.4
										EMPA vs. CD	2.44E-03	-0.59	
PC 35:1*	PC 17:0_18:1	C43H84NO8P	773.5935	818.5883	818.5917	-4.2	8.2	[M+HCOO]-	2.44E-06	FFD vs. CD	5.10E-06	2.83	5.15
										EMPA vs. CD	2.20E-05	2.70	
PC 35:2	PC 17:0_18:2	C43H82NO8P	771.5778	816.5724	816.5760	-4.4	7.2	[M+HCOO]-	7.32E-11	FFD vs. CD	4.10E-10	2.29	8.93
										EMPA vs. CD	4.70E-10	2.28	
PC 36:3*	PC 16:0_20:3	C44H82NO8P	783.5778	828.5679	828.5760	-9.8	7.6	[M+HCOO]-	1.11E-12	FFD vs. CD	2.00E-11	2.92	5.03
										EMPA vs. CD	3.10E-12	3.05	
	PC 18:1_18:2	C44H82NO8P	783.5778	828.5641	828.5760	-14.4	7.1	[M+HCOO]-	1.53E-04	FFD vs. CD	1.30E-03	0.32	4.56
										EMPA vs. CD	2.20E-04	0.37	
PC 36:4	PC 18:2_18:2	C44H80NO8P	781.5622	826.5542	826.5600	-7.0	5.6	[M+HCOO]-	9.08E-08	FFD vs. CD	1.70E-07	-3.71	1.83
										EMPA vs. CD	1.50E-06	-2.34	
PC 38:4*	PC 18:1_20:3	C46H84NO8P	809.5935	854.5861	854.5719	16.6	7.2-7.8	[M+HCOO]-	6.01E-04	FFD vs. CD	5.14E-03	1.39	18.0
										EMPA vs. CD	7.30E-04	1.58	
PC 38:6	PC 18:2_20:4	C46H80NO8P	805.5622	850.5668	850.5604	7.6	5.4	[M+HCOO]-	1.38E-05	FFD vs. CD	5.20E-05	-1.23	15.5
										EMPA vs. CD	4.80E-05	-1.24	
PC 42:10	PC 42:10	C50H80NO8P	853.5621	898.5579	898.5604	-2.7	4.8	[M+HCOO]-	4.28E-06	FFD vs. CD	6.50E-03	-0.45	15.5
										EMPA vs. CD	2.70E-06	-1.10	
PE 34:3	PE 16:1_18:2	C39H72NO8P	713.4996	712.4883	712.4923	-5.6	5.7	[M-H]-	4.44E-09	FFD vs. CD	8.80E-09	-1.73	9.24
										EMPA vs. CD	8.70E-08	-1.35	
PE 36:5	PE 16:1_20:4	C41H72NO8P	737.4996	736.4871	736.4923	-7.1	5.5	[M-H]-	1.00E-05	FFD vs. CD	9.10E-06	-0.80	4.76
										EMPA vs. CD	4.20E-04	-0.54	
PE 40:4	PE 18:0_22:4	C45H82NO8P	795.5778	794.5673	794.5705	-4.1	8.4	[M-H]-	1.30E-07	FFD vs. CD	2.50E-06	-1.85	16.6
										EMPA vs. CD	2.20E-07	-2.73	
PE 40:8	PE 20:4_20:4	C45H74NO8P	787.5152	786.5060	786.5079	-2.4	5.4	[M-H]-	2.00E-04	FFD vs. CD	1.03E-03	-0.48	14.3

	PE 18:2_22:6												
										EMPA vs. CD	3.80E-04	-0.54	
PI 34:2*	PI 34:2	C43H79O13P	834.5258	833.5138	833.5186	-5.8	4.9	[M-H]-	9.39E-11	FFD vs. CD	6.20E-10	-4.78	3.75
										EMPA vs. CD	5.10E-10	-5.28	
PI 36:3	PI 36:3	C45H81O13P	860.5415	859.5332	859.5342	-1.1	5.7	[M-H]-	2.92E-09	FFD vs. CD	1.70E-08	3.04	16.9
										EMPA vs. CD	1.50E-08	3.05	
PI 38:3*	PI 18:0_20:3	C47H85O13P	888.5728	887.5631	887.5655	-2.7	7.2	[M-H]-	3.01E-12	FFD vs. CD	9.90E-12	3.56	5.13
										EMPA vs. CD	4.20E-11	3.46	
PG 34:2	PG 34:2	C40H75O10P	746.5098	745.4998	745.5025	-3.6	5.3	[M-H]-	6.04E-07	FFD vs. CD	2.10E-06	-3.05	4.81
										EMPA vs. CD	3.30E-06	-2.78	
PG 36:4	PG 36:4	C42H75O10P	770.5098	769.5002	769.5025	-3.1	4.5	[M-H]-	7.33E-05	FFD vs. CD	2.90E-04	-1.89	17.2
										EMPA vs. CD	2.00E-04	-2.03	
PG 38:4	PG 38:4	C44H79O10P	798.5410	797.5300	797.5338	-4.8	5.0	[M-H]-	6.63E-09	FFD vs. CD	1.40E-07	4.07	5.12
										EMPA vs. CD	1.20E-08	4.27	
PG 38:6	PG 38:6	C44H75O10P	794.5097	793.4993	793.5025	-4.1	4.1	[M-H]-	3.09E-04	FFD vs. CD	3.00E-04	5.81	2.52
										EMPA vs. CD	5.40E-03	5.40	
PG 38:7	PG 16:1_22:6	C44H73O10P	792.4941	791.4835	791.4869	-4.3	3.4	[M-H]-	2.98E-08	FFD vs. CD	1.90E-07	2.07	12.4
										EMPA vs. CD	1.20E-07	2.11	
PS 40:6	PS 18:0_22:6	C46H78NO10P	835.5363	834.5259	834.5291	-3.9	6.1	[M-H]-	4.40E-06	FFD vs. CD	4.90E-03	-0.44	1.78
										EMPA vs. CD	2.70E-06	-1.01	
Cer 42:2;O2	Cer 42:2;O2	C48H91NO8	809.6745	646.6110	646.6144	-5.3	9.5	[M-H]-	3.12E-04	FFD vs. CD	1.29E-03	-0.57	6.18
										EMPA vs. CD	6.50E-04	-0.62	
Common lipids observed in the comparison FFD+EMPA male vs. FFD+EMPA female (in the serum)													
LPC 20:5	LPC 20:5	C28H48NO7P	541.3168	586.3089	586.3150	-10.4	0.9	[M+HCOO]-	5.34E-05	FFD+EMPA male vs. female		-0.74	7.14
PC 34:1	PC 16:0_18:1	C42H82NO8P	759.5778	804.5685	804.5760	-9.3	7.9	[M+HCOO]-	8.71E-04	FFD+EMPA male vs. female		0.72	6.27
PC 35:1	PC 17:0_18:1	C43H84NO8P	773.5935	818.5869	818.5917	-5.9	8.2	[M+HCOO]-	7.04E-05	FFD+EMPA male vs. female		0.56	10.3
PC 36:3	PC 18:1_18:2	C44H82NO8P	783.5778	828.5651	828.5760	-13.1	7.0	[M+HCOO]-	3.12E-04	FFD+EMPA male vs. female		0.35	8.22
PC 36:4	PC 16:0_20:4	C44H80NO8P	781.5622	826.5541	826.5600	-7.1	6.3	[M+HCOO]-	7.34E-05	FFD+EMPA male vs. female		0.39	5.87
PC 37:1	PC 18:1_19:0	C45H88NO8P	801.6247	846.6216	846.6230	-1.6	8.6	[M+HCOO]-	2.59E-04	FFD+EMPA male vs. female		1.28	7.74
PC 37:2	PC 18:2_19:0	C45H86NO8P	799.6091	844.6044	844.6073	-3.4	8.3	[M+HCOO]-	4.96E-04	FFD+EMPA male vs.		1.05	10.2

										female		
PC 38:2	PC 18:0_20:2	C46H88NO8P	813.6247	858.6278	858.623	5.6	8.4	[M+HCOO]-	6.00E-05	FFD+EMPA male vs. female	1.12	23.4
PC 38:4	PC 18:0_20:4	C46H84NO8P	809.5935	854.5775	854.5917	-16.6	8.0	[M+HCOO]-	1.12E-04	FFD+EMPA male vs. female	0.64	6.49
PC 38:5	PC 18:1_20:4	C46H82NO8P	807.5778	852.5665	852.5760	-11.1	6.5	[M+HCOO]-	1.62E-04	FFD+EMPA male vs. female	0.63	6.79
	PC 16:0_22:5<br>PC 18:0_20:5	C46H82NO8P	807.5778	852.5665	852.5760	-11.1	6.9	[M+HCOO]-	2.37E-04	FFD+EMPA male vs. female	0.34	5.24
PC 38:7	PC 16:1_22:6	C46H78NO8P	803.5465	848.5377	848.5447	-8.2	4.8	[M+HCOO]-	8.09E-06	FFD+EMPA male vs. female	1.31	20.5
PC 40:7	PC 18:1_22:6	C48H82NO8P	831.5778	876.5716	876.5760	-5.1	6.1	[M+HCOO]-	1.05E-05	FFD+EMPA male vs. female	0.42	6.87
PC 40:8	PC 18:2_22:6<br>PC 20:4_20:4	C48H80NO8P	829.5622	874.5510	874.5604	-10.7	5.0	[M+HCOO]-	1.51E-05	FFD+EMPA male vs. female	0.68	9.93
HexCer 40:1;O2	HexCer 40:1;O2	C46H89NO8	783.6588	828.6518	828.6570	-6.3	9.0	[M+HCOO]-	5.52E-05	FFD+EMPA male vs. female	1.17	8.80
Common lipids observed in the comparison between FFD+EMPA male vs. FFD+EMPA female (in the liver)												
PS 38:4	PS 18:0_20:4	C44H78NO10P	811.5363	810.5292	810.5291	0.1	6.4	[M-H]-	8.09E-04	FFD+EMPA male vs. female	-0.66	4.28
Cer 36:1;O2	Cer 36:1;O2	C36H71NO3	565.5434	610.5403	610.5416	-2.1	8.5	[M+HCOO]-	4.03E-05	FFD+EMPA male vs. female	-1.77	6.51
Cer 40:0;O2	Cer 40:0;O2	C40H81NO3	623.6216	668.6086	668.6086	0.0	9.5	[M+HCOO]-	6.14E-05	FFD+EMPA male vs. female	0.61	4.71
SM 32:1;O2	SM 32:1;O2	C37H75N2O6P	674.5363	719.5309	719.5345	-5.0	4.6	[M+HCOO]-	1.65E-04	FFD+EMPA male vs. female	-1.08	4.02
SM 33:1;O2	SM 33:1;O2	C38H77N2O6P	688.5519	733.5473	733.5501	-3.8	5.3	[M+HCOO]-	1.80E-05	FFD+EMPA male vs. female	-1.16	5.24

*Common lipids for the comparisons between FFD vs. CD and EMPA vs. CD in both serum and liver tissue.

a: unadjusted p-values.

There are not common lipids for the comparison between FFD+EMPA male vs. FFD+EMPA female in the serum and liver.

Abbreviations: CD, chow diet; Cer, ceramide; EMPA, empagliflozin; FFD, fast food diet; HexCer, hexosylceramide; log_2FC, log_2(Fold Change); LPC, lysophosphatidylcholine; LPE, lysophosphatidylethanolamine; LPI, lysophosphatidylinositol; PC, phosphatidylcholine; PE, phosphatidylethanolamine; PG, phosphatidylglycerol; PI, phosphatidylinositol; PS, phosphatidylserine; RT min, retention time in minutes; SM, sphingomyelin; Δ ppt, delta parts per million.
