# Supplementary material for: Serum and Liver Lipidome Following Empagliflozin Administration for Six Months in a Fast Food Diet Mouse Model
Source: Int J Mol Sci. 2025 Sep 23;26(19):9273. doi: 10.3390/ijms26199273 (PMC12524644; doi:10.3390/ijms26199273)
Supplement: Supplementary file 1 [file ijms-26-09273-s001.zip › Polyzos_Table S3.pdf]

Table S3.Comparisons of lipid classes across groups and sex subgroups.*

		FFD	EMPA	CD	p-value for trend	p-value for pairwise comparison FFD vs. EMPA	FFD vs. CD	EMPA vs. CD
Serum								
**SUM LPC**	total	15555105±1691839	11362653±2010313	12223423±1424259	<0.001	<0.001	0.006	1.00
	male	16095866 ±2157896	11411409±2883318	12443071±405239	0.04	0.05	0.19	1.00
	female	15014345 ±1114646	11313897±1053553	12003775±2182277	0.02	0.02	0.07	1.00
**SUM LPE**	total	2673615±354148	1946840±621900	3614942±890622	<0.001	0.10	0.04	<0.001
	male	2537025±417649	2249423±467155	4265191±805965	0.003	1.00	0.01	0.004
	female	2810206±261662	1644257±663367	2964693±254888	0.008	0.02	1	0.02
**SUM PC**	total	90229201±15453059	102183156±29299562	41580812±9251481	0.001	1.00	0.008	0.003
	male	100942807±11453807	129404661±4608196	49655877±3302917	<0.001	0.003	<0.001	<0.001
	female	79515595±10950790	74961652±2412576	33505746±2728634	<0.001	1.00	<0.001	<0.001
**SUM PI**	total	3318098±710416	2991327±306911	1395553±503786	<0.001	0.71	<0.001	<0.001
	male	3456952±977299	3001082±168310	1368580±602913	0.01	1.00	0.01	0.04
	female	3179245±413626	2981572±437270	1422525±518473	0.002	1.00	0.003	0.006
**SUM Sphingolipids**	total	2328296±882026	2699156±1019935	801970±123308	0.001	1.00	0.008	0.001
	male	2253579±392775	2172561±259581	2179883±80793	<0.001	0.08	<0.001	<0.001
	female	3247808±313363	3312081±573683	2931450±363512	0.05	-	-	-
Liver								
**SUM LPC**	total	26166147±7571485	20039133±6179831	53520633±7621144	<0.001	0.30	<0.001	<0.001
	male	28166179±7176041	15462365±1229625	48366828±6115139	<0.001	0.03	0.004	<0.001
	female	24166114±8461756	24615901±5633879	57385987±6689599	<0.001	1.00	<0.001	<0.001
**SUM LPE**	total	1532583±468337	1225833±735119	3947128±885720	<0.001	1.00	<0.001	<0.001
	male	1525106±497419	675859±168125	3473758±1082199	0.001	0.28	0.01	0.001
	female	1540060±514022	1775808±652745	4302155±629361	<0.001	1.00	<0.001	<0.001
**SUM LPI**	total	1641417±296366	1337799±480965	713616.6±121497	<0.001	0.27	<0.001	0.006
	male	1679043±422268	976469.5±269586	639546±130908	0.006	0.04	0.007	0.59
	female	1603791±151190	1699129±344863	769169±92180	<0.001	1	0.002	<0.001
**SUM PC**	total	48257234±3851420	47744625±6239173	38804764±5674441	0.004	1.00	0.008	0.01

	male	47056416±4281977	45151118±2778665	41982819±6948641	0.51	-	-	-
	female	49458052±3525731	50338132±8072955	36421223±3812404	0.01	1.00	0.03	0.02
**SUM PE**	total	2868728±422754	3105267±477726	5877045±398502	<0.001	0.87	<0.001	<0.001
	male	3076035±511201	2795903±143311	5966626±499414	<0.001	1	<0.001	<0.001
	female	2661421±202699	3414632±506717	5809859±370525	<0.001	0.06	<0.001	<0.001
**SUM PI**	total	11634876±969265	10974783±1627533	2291547±844873	<0.001	0.87	<0.001	<0.001
	male	11413275±929800	10016050±1477580	2587659±1047861	0.03	1	0.008	0.003
	female	11856477±1093904	11933517±1243511	2069464±736322	<0.001	1	<0.001	<0.001
**SUM PG**	total	1658543±440040	1729002±418148	1963791±628665	0.48	-	-	-
	male	1610218±499084	1626142±615103	1921220±431352	0.71	-	-	-
	female	1706868±443287	1831863±37588	1995719±814386	0.75	-	-	-
**SUM PS**	total	2800760±745877	2441288±534950	3442023±229333	0.008	0.64	0.11	0.007
	male	2198324±457995	2165378±363411	3291908±29276	0.006	1	0.012	0.01
	female	3403196±347185	2717198±576768	3554609±255304	0.04	0.133	1	0.057
**SUM Sphingolipids**	total	2750693±625001	2742321±735478	2609350±479200	0.89	-	-	-
	male	2253579±392775	2172561±259581	2179883±80793	0.91	-	-	-
	female	3247808±313363	3312081±573683	2931450±363512	0.44	-	-	-

*: Data represent the summed intensities of lipids per class.

Abbreviations: CD, Chow diet; EMPA, Empagliflozin group; FFD, Fast food diet group; LPC, Lysophosphatidylcholine; LPE, Lysophosphatidylethanolamine; LPI, Lysophosphatidylinositol; PC, Phosphatidylcholine; PE, Phosphatidylethanolamine; PG, Phosphatidylglycerol; PI, Phosphatidylinositol; PS, Phosphatidylserine; SUM, Sum of measured lipids.
