# Supplementary material for: Serum and Liver Lipidome Following Empagliflozin Administration for Six Months in a Fast Food Diet Mouse Model
Source: Int J Mol Sci. 2025 Sep 23;26(19):9273. doi: 10.3390/ijms26199273 (PMC12524644; doi:10.3390/ijms26199273)
Supplement: Supplementary file 1 [file ijms-26-09273-s001.zip › Polyzos_Table S4.pdf]

Table S4. Composition of diets.

Nutrients	Diet	
	**FFD**	**CD**
Protein (% weight)	17.3	19.0
Fat (% weight)	21.1	3.3
Carbohydrates (% weight)	48.7	40.5
Cholesterol (% weight)	0.21	0.014
Fiber (% weight)	5.0	4.9
Fructose in water (g/l)	23.1	0.0
Glucose in water (g/l)	18.9	0.0

Abbreviations: CD, chow diet; FFD, fast food diet.
