# Supplementary figures and images for: Serum and Liver Lipidome Following Empagliflozin Administration for Six Months in a Fast Food Diet Mouse Model
Source: Int J Mol Sci. 2025 Sep 23;26(19):9273. doi: 10.3390/ijms26199273 (PMC12524644; doi:10.3390/ijms26199273)

## Slide 1
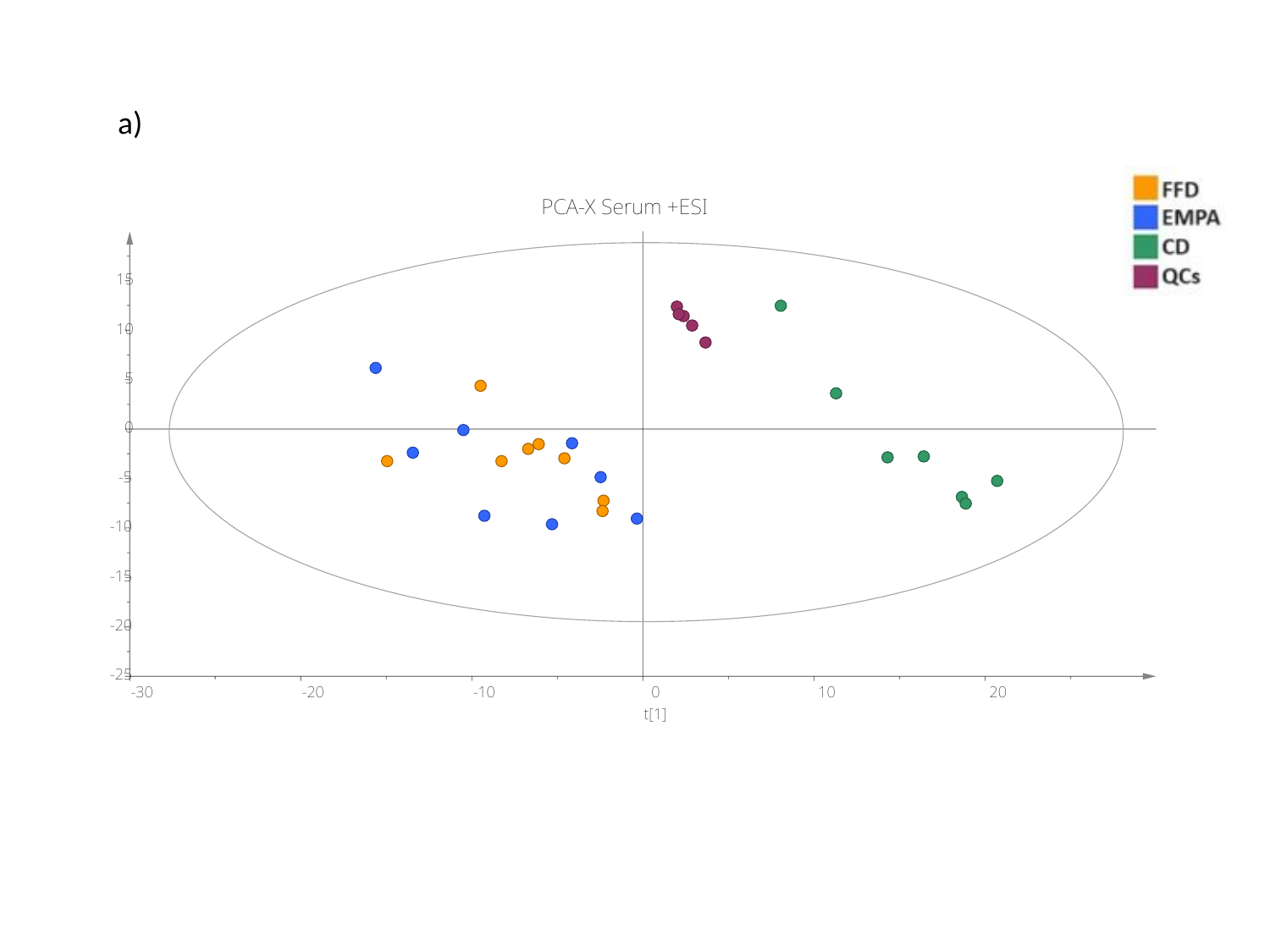

a)

## Slide 2
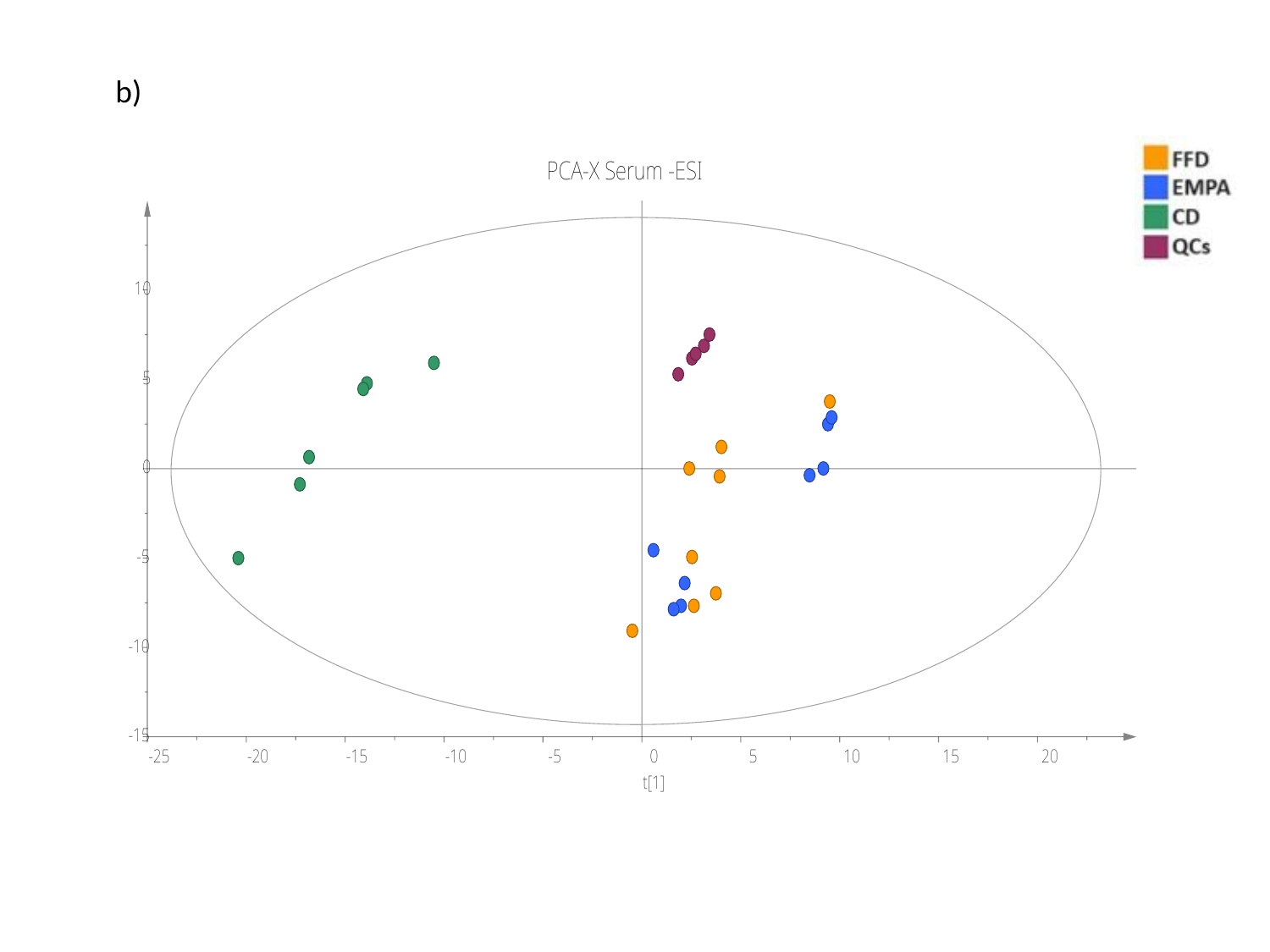

b)

## Slide 3
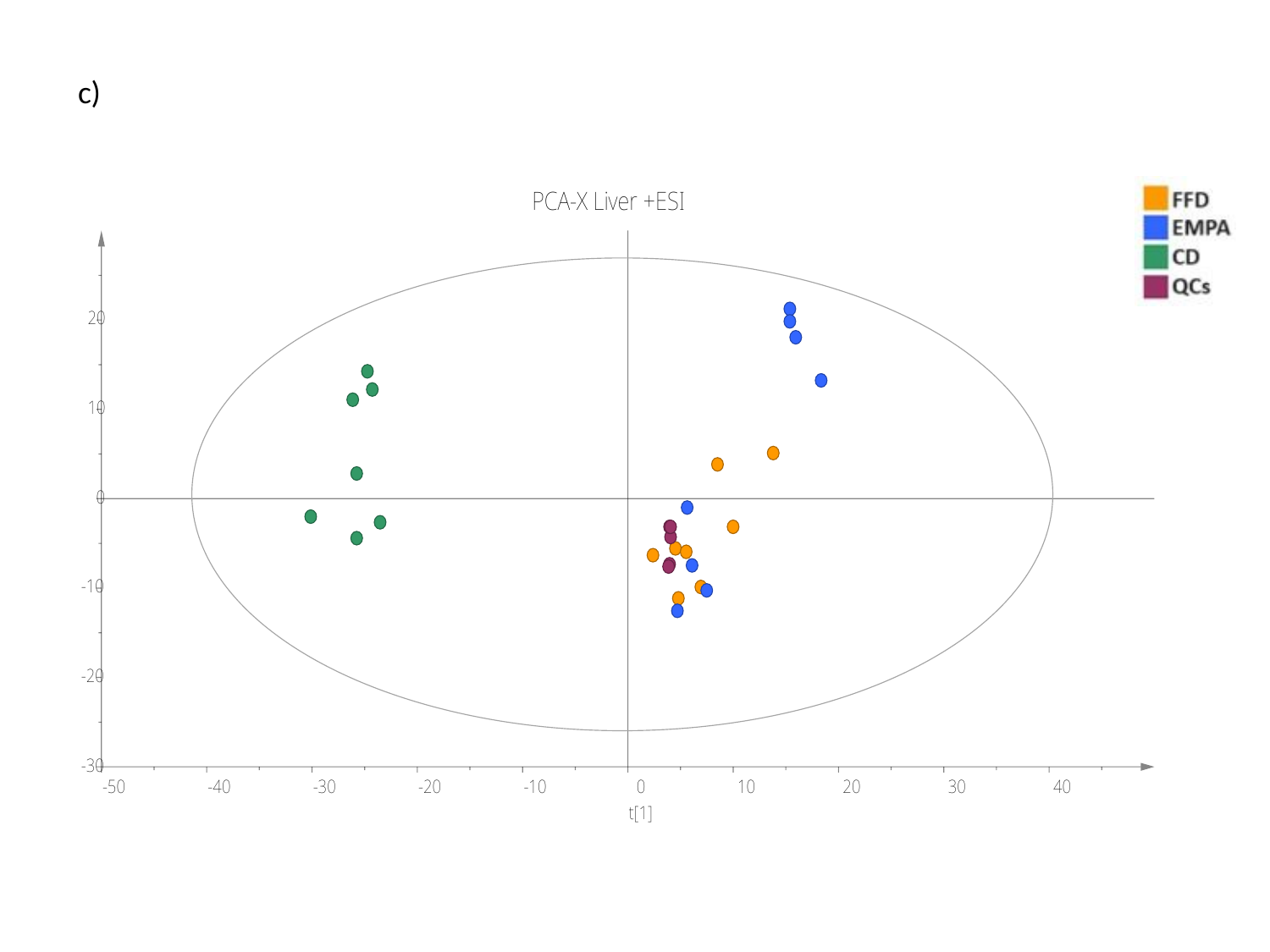

c)

## Slide 4
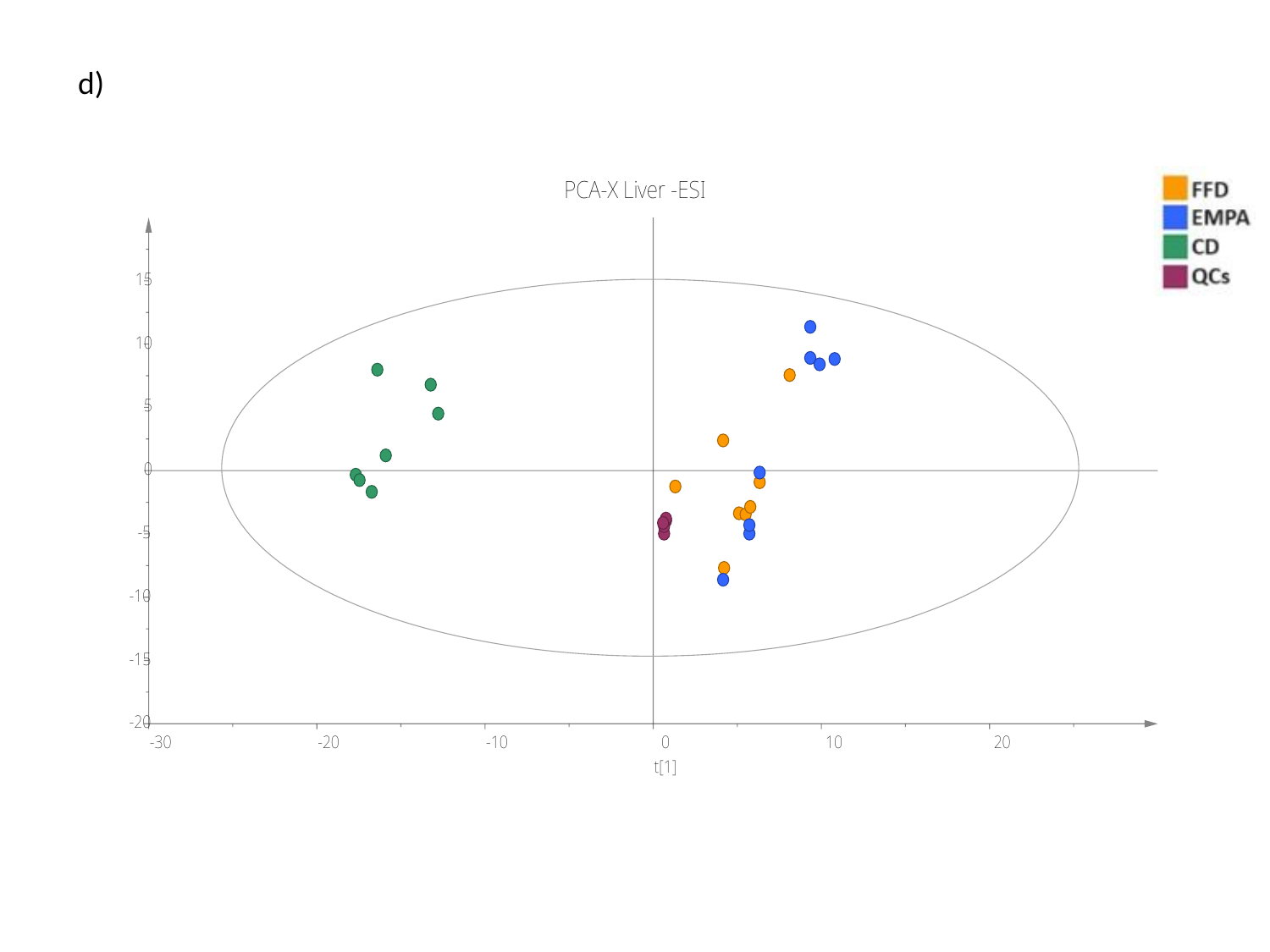

d)
FFD
EMPA
CD
QCs

Supplement: Supplementary file 1 [file ijms-26-09273-s001.zip › Polyzos_Figure S1.pptx]

## Slide 1
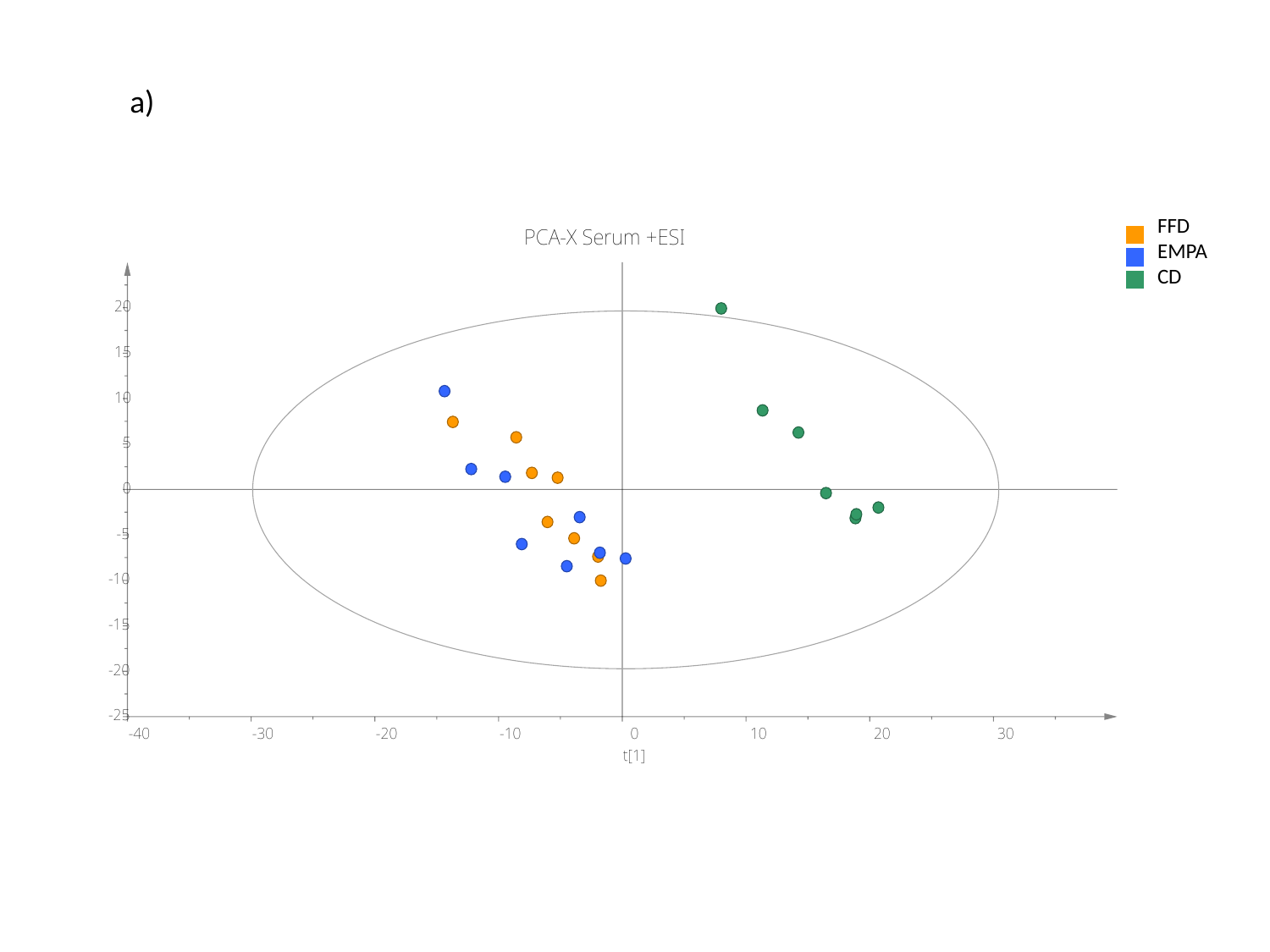

a)
FFD
EMPA
CD

## Slide 2
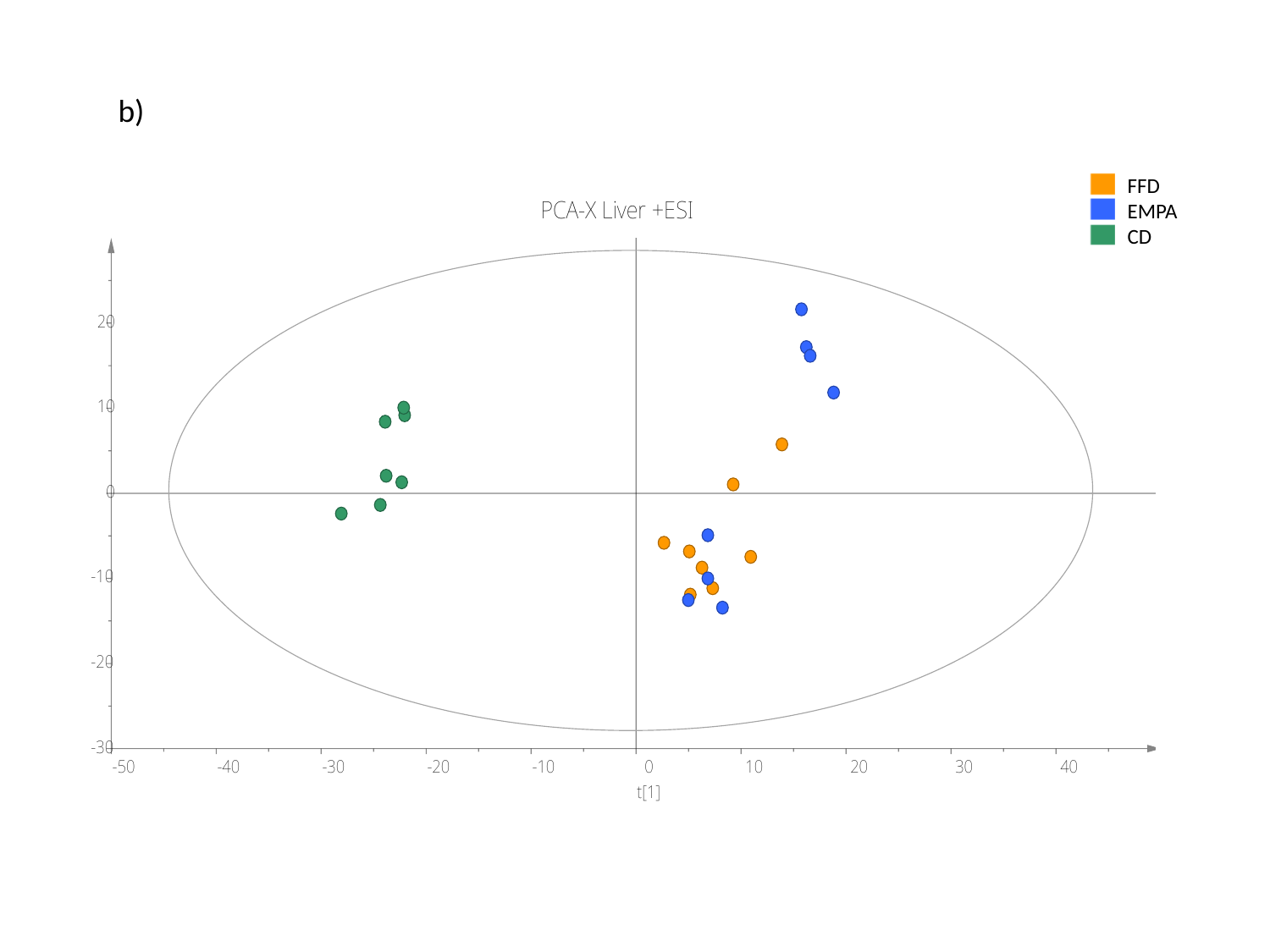

b)
FFD
EMPA
CD

## Slide 3
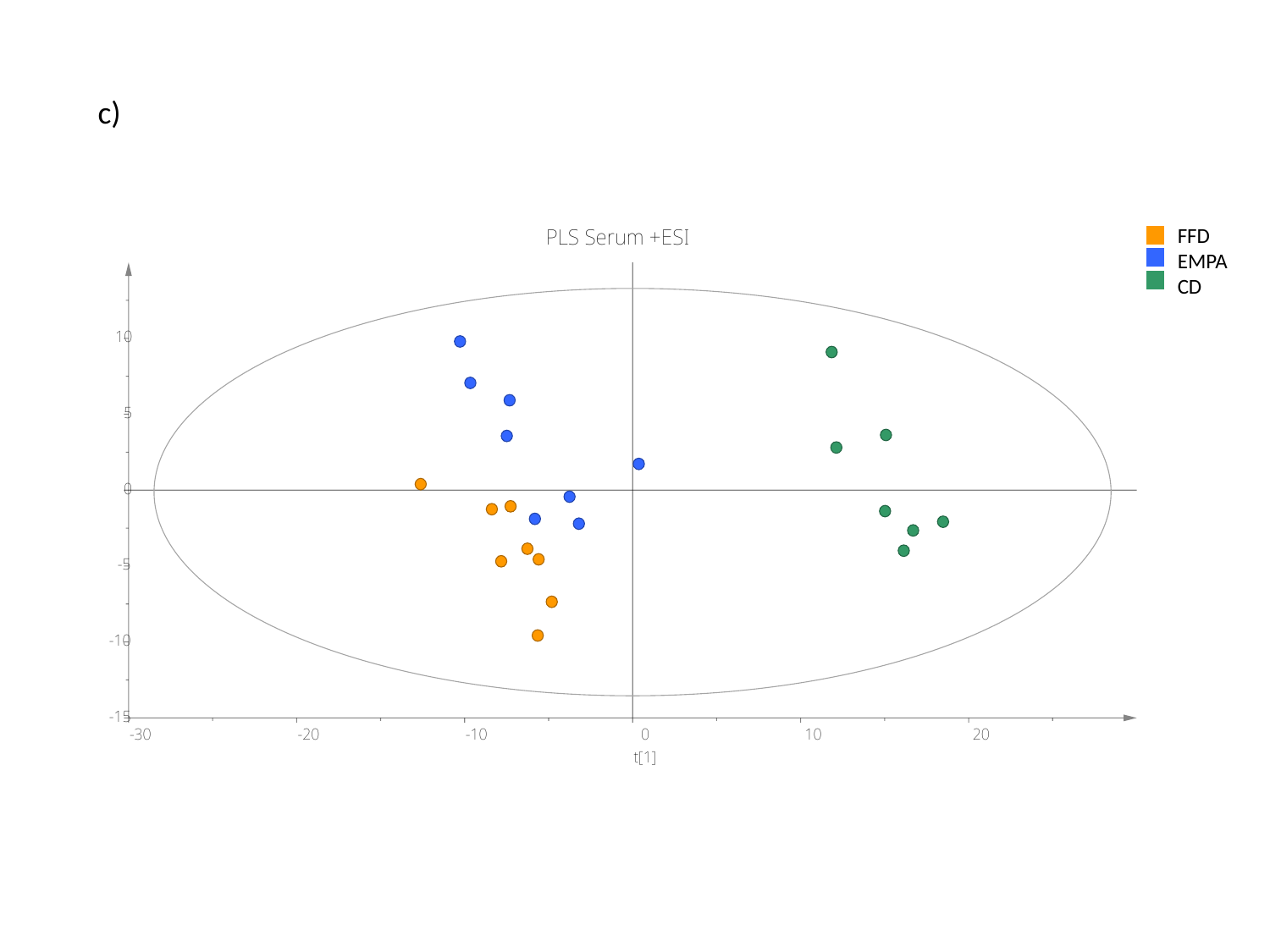

c)
FFD
EMPA
CD

## Slide 4
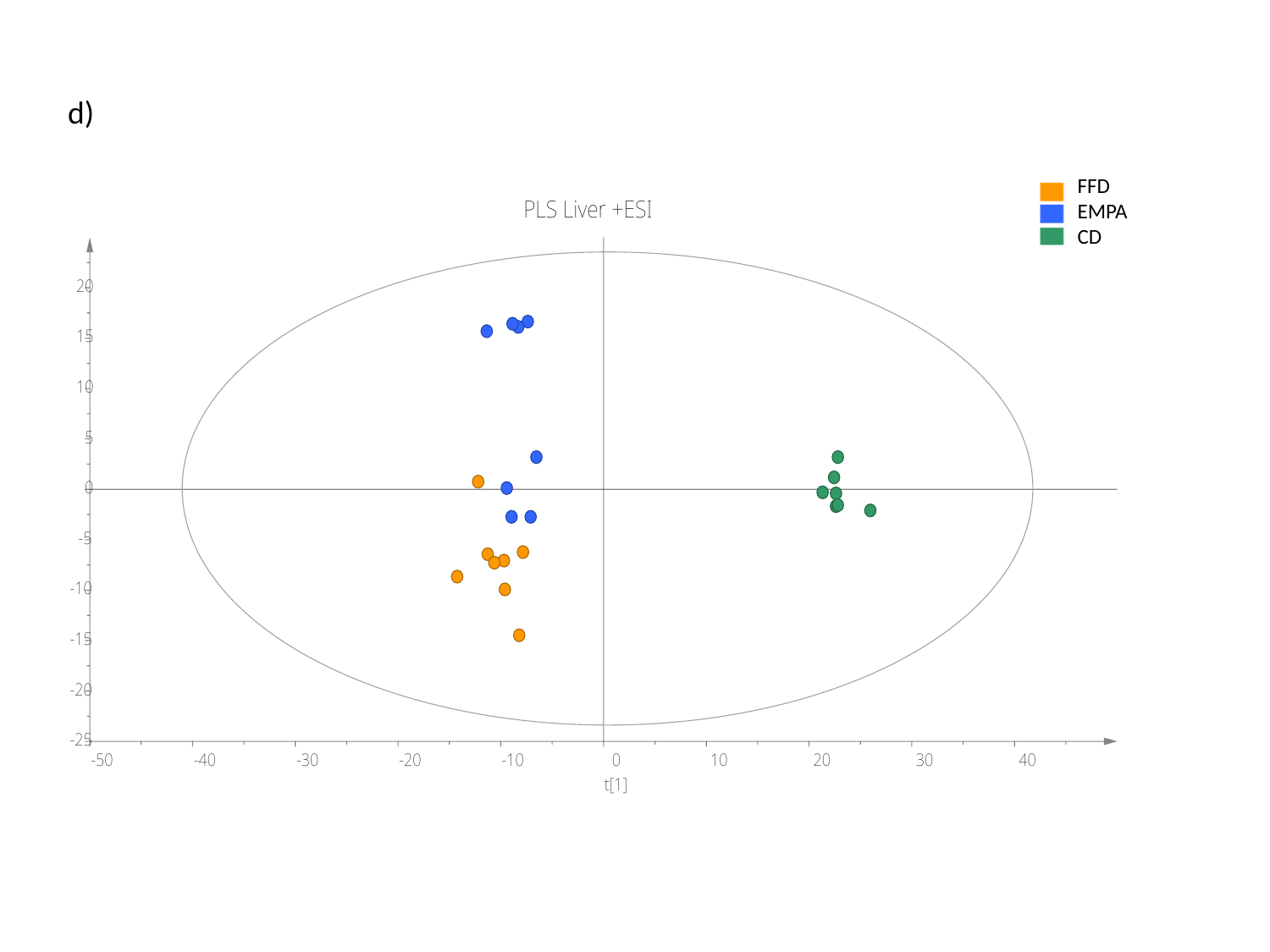

d)
FFD
EMPA
CD

Supplement: Supplementary file 1 [file ijms-26-09273-s001.zip › Polyzos_Figure S2.pptx]

## Slide 1
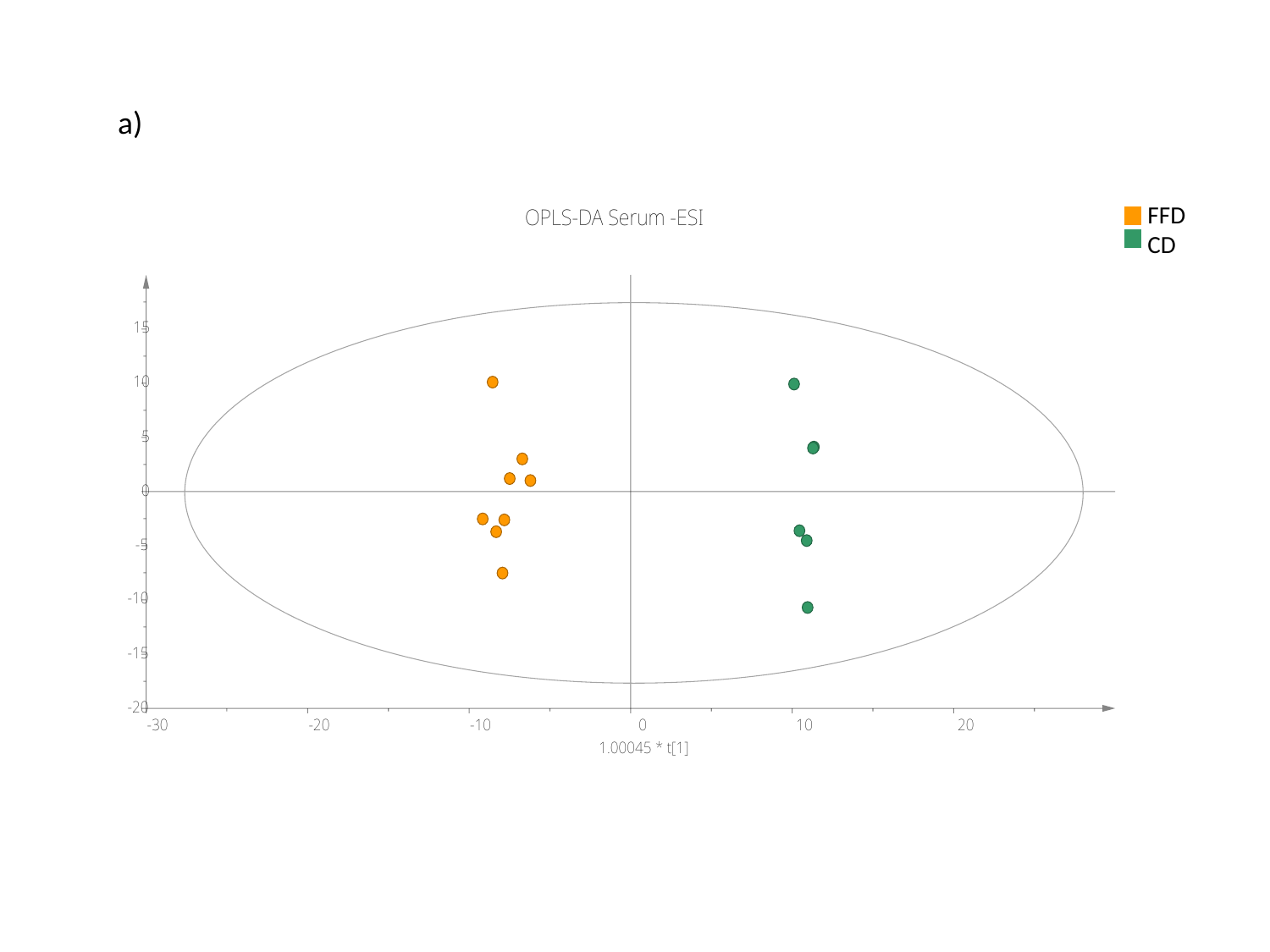

a)
FFD
CD

## Slide 2
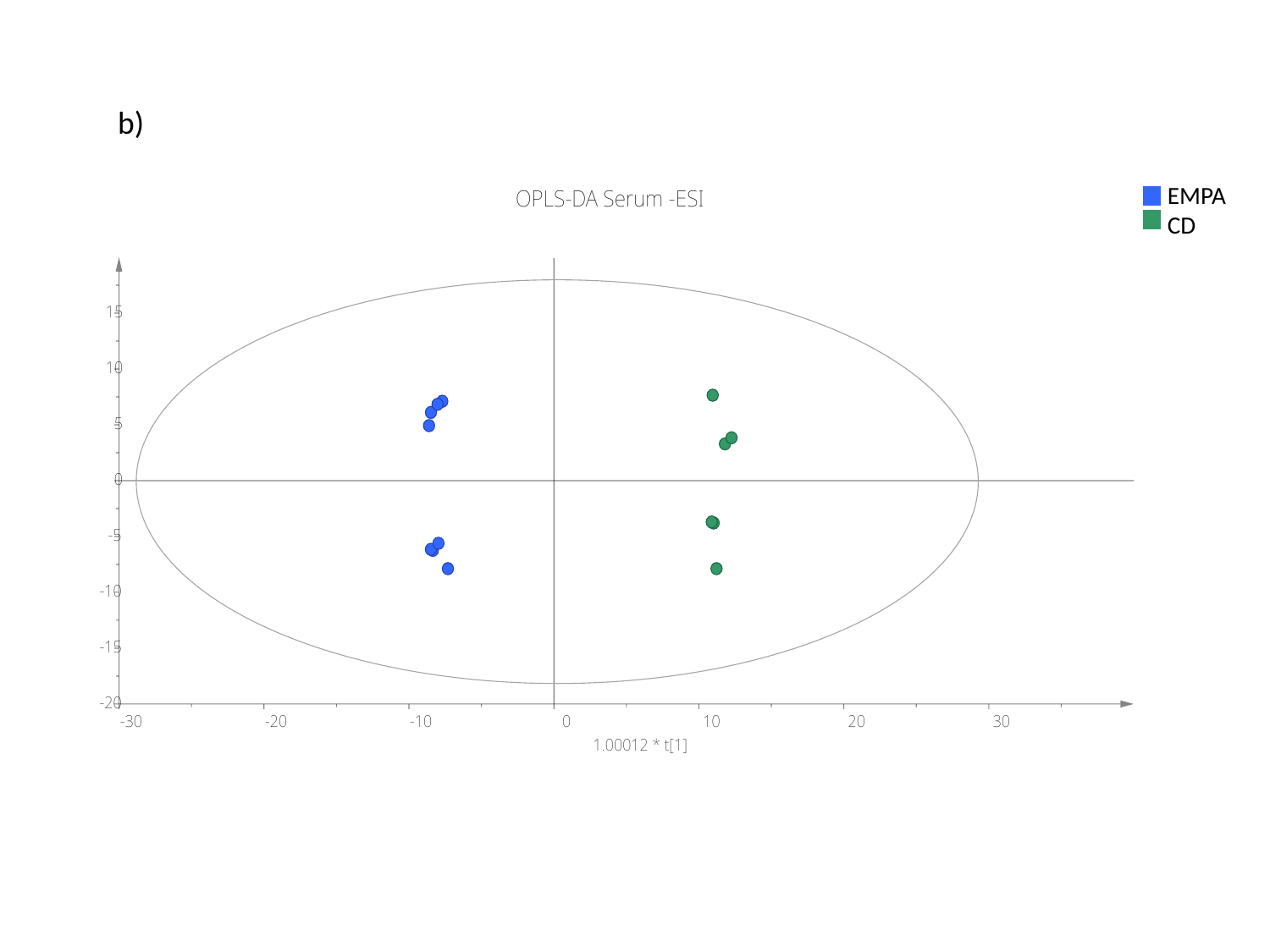

b)
EMPA
CD

## Slide 3
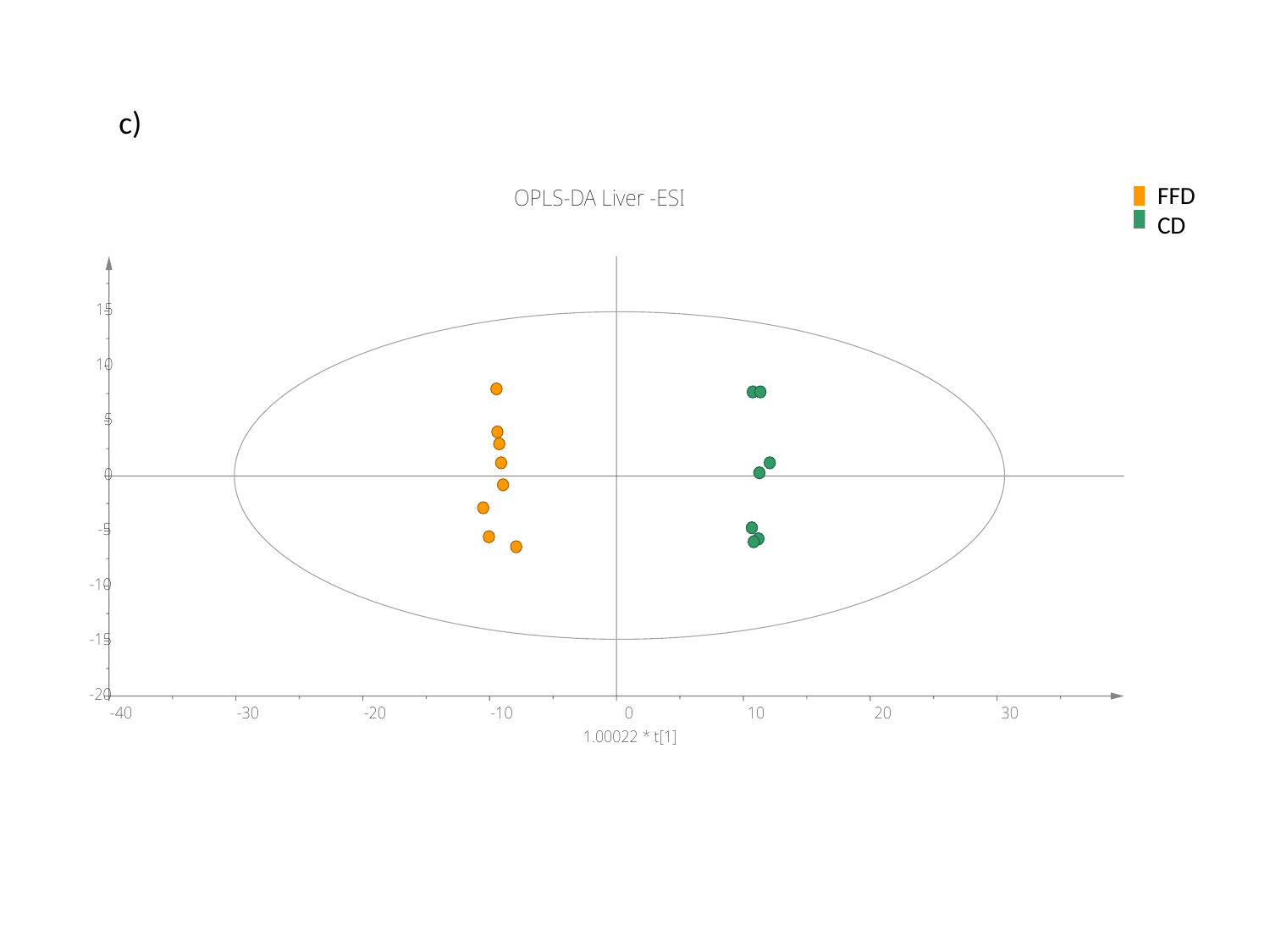

c)
FFD
CD

## Slide 4
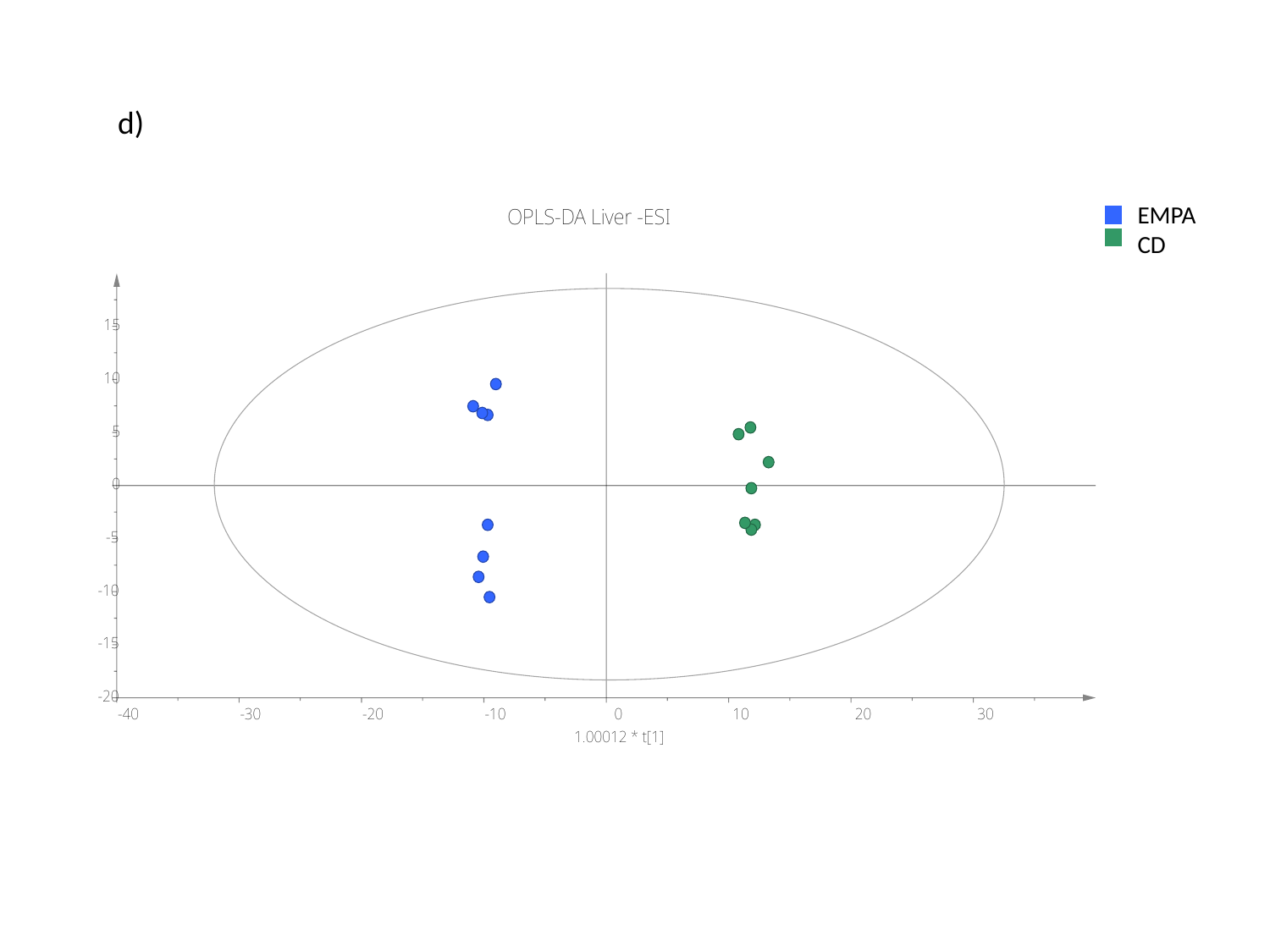

d)
EMPA
CD

Supplement: Supplementary file 1 [file ijms-26-09273-s001.zip › Polyzos_Figure S3.pptx]
